# Forecasting global and multi-level thermospheric neutral density and ionospheric electron content by tuning models against satellite-based accelerometer measurements

**DOI:** 10.1038/s41598-022-05952-y

**Published:** 2022-02-08

**Authors:** Ehsan Forootan, Mona Kosary, Saeed Farzaneh, Timothy Kodikara, Kristin Vielberg, Isabel Fernandez-Gomez, Claudia Borries, Maike Schumacher

**Affiliations:** 1 Geodesy Group, Department of Planning, Aalborg University, Rendburggade 14, 9000 Aalborg, Denmark; 2 School of Surveying and Geospatial Engineering, College of Engineering, University of Tehran, P.O. Box 113654563, Tehran, Iran; 3 Institute of Solar-Terrestrial Physics, German Aerospace Center (DLR), Kalkhorstweg 53, 17235 Neustrelitz, Germany; 4 Institute of Geodesy and Geoinformation, University of Bonn, Nussallee 17, 53115 Bonn, Germany

**Keywords:** Atmospheric dynamics, Space physics

## Abstract

Global estimation of thermospheric neutral density (TND) on various altitudes is important for geodetic and space weather applications. This is typically provided by models, however, the quality of these models is limited due to their imperfect structure and the sensitivity of their parameters to the calibration period. Here, we present an ensemble Kalman filter (EnKF)-based calibration and data assimilation (C/DA) technique that updates the model’s states and simultaneously calibrates its key parameters. Its application is demonstrated using the TND estimates from on-board accelerometer measurements, e.g., those of the Gravity Recovery and Climate Experiment (GRACE) mission (at $\sim 410$ km altitude), as observation, and the frequently used empirical model NRLMSISE-00. The C/DA is applied here to re-calibrate the model parameters including those controlling the influence of solar radiation and geomagnetic activity as well as those related to the calculation of exospheric temperature. The resulting model, called here ‘C/DA-NRLMSISE-00’, is then used to now-cast TNDs and individual neutral mass compositions for 3 h, where the model with calibrated parameters is run again during the assimilation period. C/DA-NRLMSISE-00 is also used to forecast the next 21 h, where no new observations are introduced. These forecasts are unique because they are available globally and on various altitudes (300–600 km). To introduce the impact of the thermosphere on estimating ionospheric parameters, the coupled physics-based model TIE-GCM is run by replacing the O2, O1, He and neutral temperature estimates of the C/DA-NRLMSISE-00. Then, the non-assimilated outputs of electron density (*Ne*) and total electron content (TEC) are validated against independent measurements. Assessing the forecasts of TNDs with those along the Swarm-A ($\sim 467$ km), -B ($\sim 521$ km), and -C ($\sim 467$ km) orbits shows that the root-mean-square error (RMSE) is considerably reduced by 51, 57 and 54%, respectively. We find improvement of 30.92% for forecasting *Ne* and 26.48% for TEC compared to the radio occulation and global ionosphere maps (GIM), respectively. The presented C/DA approach is recommended for the short-term global multi-level thermosphere and enhanced ionosphere forecasting applications.

## Introduction

Space weather describes physical processes caused by the Sun’s radiation of energy. The manifestations of space weather are multiple, e.g., the variations of the Earth’s magnetic field or the changing states of the upper atmosphere—between the altitude of around 100 km up to 2000 km—comprising both the thermosphere and the ionosphere. An accurate quantification of the thermospheric neutral density (TND) and thermospheric temperature has become increasingly important in the space physics and geodesy community^1^. Atmospheric drag has a direct relationship with the TND estimates and represents a considerable impact on the precise orbit determination (POD) and prediction of low Earth orbit (LEO) satellites, for example those with the altitude of less than 1000 km, as well as space debris^2^. The ionosphere variability impacts operational communications and navigation systems, thus, its now-casting and forecasting is extremely important for many applications^3–7^. Energy and momentum are transferred from the lower to the upper atmosphere and ionosphere through the generation and propagation of waves. These interactions make the thermosphere-ionosphere a coupled system that exhibit non-linear and complex interactions with many inherent time-scales^8^.

The accelerometer sensors on-board of satellites, which measures the non-gravitational forces acting on them, have been emerged as useful tools for estimating TND and its changes^9^. The LEO missions including the Challenging Minisatellite Payload (CHAMP, 2000–2010)^10^, the Gravity Recovery and Climate Experiment (GRACE, 2002–2017)^11^ and its Follow-On mission (GRACE-FO, launched in 2018)^12^, Gravity field and steady-state Ocean Circulation Explorer (GOCE, 2009–2013)^13^ and the European Space Agency (ESA)’s Swarm mission (Swarm- A, -B, and -C launched in 2013)^14^, are applied by various centers to estimate TNDs, e.g., from the Delft Technical University^15^, European Space Agency^16^, Mehta et al.^17^, and Vielberg et al.^18^. However, these measurements are available only along-track of these missions, thus, their application for studying global and multi-level neutral density changes in the thermosphere is limited. In this study, we will provide a technique to address this limitation, and we will demonstrate the benefits of accelerometer-based TND estimates for global and multi-(vertical)level thermosphere and ionosphere studies.

Empirical and physical models are developed to address the demands for precise estimation of thermospheric variables such as the TND and its individual neutral compositions, thermospheric temperature, as well as ionospheric parameters such as total electron content (TEC), the three-dimensional electron density, and its evolution in time (fourth dimension)^19^. Physical models are based on physical laws and principles such as continuity, energy and momentum equations and require the solution of partial differential equations^20,21^, while empirical models are driven by fitting mathematical equations to actual measurements, as provided from one or more observation systems, during an appropriate time period^22–25^. The current status of measuring and modeling techniques related to thermosphere and ionosphere is reviewed by Palmroth et al. (2021)^26^.

A logical step to make the best use of observations and models can be realized through the mathematical merging frameworks that build a connection between them. For example, (1) correction fields are applied as a ratio of TNDs from LEO satellites and those of models^27–33^. However, this method can only be used for reanalyzing and now-casting the thermosphere, and the reliability of these ratio fields, derived by a limited number of satellite tracks, might be treated with caution. (2) Statistical data-model integration frameworks apply decomposition techniques^34^ to extract the dominant structures of the upper atmosphere dynamics. Then, predicting steps are performed using state-space techniques such as the Kalman filter (KF)^35^. Previous studies^36–40^ successfully applied this approach during geomagnetic storms because the pronounced temporal and spatial changes during these events enhance the model-data integration. This technique might be less efficient during calm periods, where the level of uncertainties of models and data is comparable. (3) Sequential data assimilation (DA) techniques are found to be efficient for merging observations and model outputs, while decreasing the model uncertainties^41–45^. However, DA techniques mostly focus on updating the model states and they are not very efficient to extend the along-track measurements to cover the whole globe. Besides, they are not very efficient for forecasting. (4) The calibration and data assimilation (C/DA) can provide the opportunity to update the model’s states (similar to DA) and can simultaneously calibrate the model’s key parameters^46,47^. The advantage of this approach is that the calibrated parameters can be used to simulate TNDs in locations outside of the orbital tracks of LEO satellite, or to forecast TNDs.

The methodology of this study follows that of Forootan et al.^47^, categorized as (4), who proposed a C/DA method based on the sequential ensemble Kalman filter (EnKF) to calibrate the NRLMSISE-00 model^22^. In their study, CHAMP and GRACE TND estimates, normalised at 400 km altitude, were used as observation. They applied C/DA to estimate the daily calibrated key parameters of NRLMSISE-00, which referred to the solar radiation, geomagnetic activity, and calculation of the exospheric temperature. Then the calibrated parameters were used to forecast the global TND fields of the next day at 400 km altitude.

Here, we extend the previous studies by applying C/DA while using along-track TNDs without height-dependent normalisation, i.e., using GRACE data at its altitude, e.g., $\sim 410$ km during February 2015. The new model that contains the re-calibrated parameters through C/DA is named here as ‘C/DA-NRLMSISE-00’. The effect of this (re-)calibration is examined by running the C/DA-NRLMSISE-00 up to 21-h in forecasting-mode (i.e., without introducing new observations) and estimating the global TND as well as individual neutral mass densities covering the altitudes of 300–600 km. TND estimations from the POD analysis of Swarm data at the altitude range of 470–520 km (during February 2nd–28th, 2015) are then used for validation.

To investigate the thermosphere-ionosphere interactions, the impact of the C/DA-derived neutral mass density estimations is studied on the ionosphere parameters, e.g., electron density and total electron content (TEC). This investigation was missing in most of the existing literature. For this, the physics-based model TIE-GCM^20^ is chosen as basis. This model was run twice, i.e., first with its original primary history fields. In the second run, we replaced some of the history fields, which are mass mixing ratios of atomic oxygen (O), molecular oxygen ($\mathrm{O}_{2}$), and helium (He), as well as the neutral temperature (TN) using the forecasting estimates derived from C/DA-NRLMSISE-00. The second run is considered to take advantage of the improved representation of thermospheric parameters thus the new runs are named ‘TIE-GCM-I’. For validation, the electron density and TEC estimates of the original TIE-GCM and TIE-GCM-I are validated against the measurements from COSMIC^48^ and the global ionosphere maps (GIM)^49^. Thus, the investigations related to the ionosphere part (i.e., during February 2015) are completely independent from the type of measurements that we used in the C/DA procedure.

## Data and model

### Space-borne thermospheric neutral density

**Figure 1:**
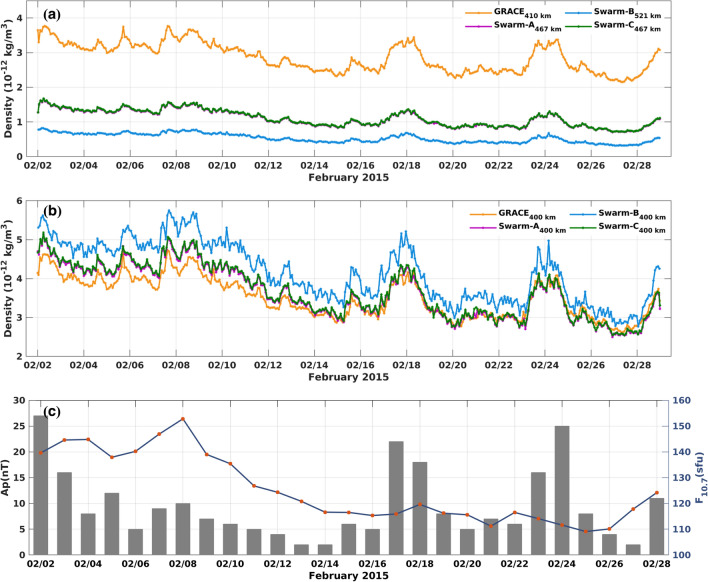
From top to bottom: (**a**) orbit-averaged TND from GRACE, Swarm-A, -B, and -C. on their original altitude. (**b**) Time series of orbit-averaged TND from GRACE, Swarm-A, -B, and -C normalised on a common altitude of 400 km. (**c**) Time series of the solar ($F_{10.7}$ ) and geomagnetic activity (*Ap*) indices during February 2015. The plots were generated using MATLAB (version R2021a, https://www.mathworks.com/).

Space-borne TND estimates, which will be used as observations in the C/DA procedure and for validation are derived from GRACE^11^ and the ESA’s Swarm mission^14^, respectively. GRACE-TNDs with 30 s sampling rate are available by Vielberg et al. (2021)^18^. The TNDs of Swarm POD (at 30-s sampling rate) are downloaded from the Delft Technical University’s website^15^. More details about these data can be found in the Supplementary file (Section S2 online). Figure 1 provides an overview of the data used in this study. Panel (a) presents time series of the mean TND estimates derived from GRACE and Swarm missions along their orbits at their original altitude during February 2015, and (b) represents them at the common altitude of 400 km. The height-dependent function and vertical profile of NRLMSISE-00 model was used to transform the TNDs to a common altitude of 400 km (see the Supplementary file Section S3 online), which makes it easier to compare the consistency of TNDs derived from GRACE and Swarm-A/-B/-C^47^. In fact, this figure demonstrates that a bias exists between the TND estimates of various satellite missions and the mean bias of $-1.67\times 10^{-13}$ kg/$\hbox {m}^3$ is estimated between the four TND estimates of Fig. 1b. Based on these results, one may conclude that the estimation of TND from Swarm-B that is on much higher altitude (on average 520 km) contains bigger biases that the other missions. Thus, comparisons with this estimation might be done with caution. It is worth mentioning that in our study the multi-mission data are not used within the C/DA and the procedure is implemented only for GRACE. But for validation we use the Swarm data while taking into the account the uncertainties that are shown in Fig. 1b. In Panel (c), the variation of solar and planetary geomagnetic activity are shown using $F_{10.7}$ and *Ap* indices, respectively.

Figure 1 indicates that the trend of changes in TNDs depends on the variation of solar activity. For example, compare the peaks of (b) and (c), where the maximum value of TNDs corresponds to the day with the high solar activity (i.e., February 8th, 2015). Sudden peaks in *Ap* for example, during February 18–19th and 23–24th, 2015 also caused changes in the magnitude of TNDs (compare the gray bars of Fig. 1c with the time series in Fig. 1b).

### Electron density profile from radio occultation measurements

To validate the ionosphere related parameters, *Ne* profiles of the original TIE-GCM model and TIE-GCM-I are compared with the COSMIC radio occultation^48^. The COSMIC constellation includes six satellites, which was launched on April 15th, 2006. Their initial orbit altitude was 500 km, but it was gradually raised to 800 km. In this study, the second-level ‘ionPrf’ data products^48^ is used, whereas their accuracy is evaluated to be around $10^{4}$–$10^{5}$ $\hbox {cm}^{-3}$, see^48^. Before assessing the TIE-GCM-I with RO data, it is necessary to apply some quality control tests on the individual ionospheric electron density profiles. For this, the least squares method is applied to fit a two-layer Chapman function to each profile^50^. This makes the best fit with RO electron density profiles at the F2 layer. In addition, we estimated the mean deviation of these profiles to quantify the effect of ionospheric plasma irregularities on the height-dependent variability of the electron density^51^.

### Global ionosphere maps (GIM)

As our second assessment, related to the ionosphere parameters, the TEC forecasts of TIE-GCM-I are compared with those of the IGS (International GNSS Service) products^49^. Since 1998, Based on GNSS dual-frequency code and phase measurements from globally distributed IGS tracking stations and seven Ionospheric Associate Analysis Centers (IAACs) have established products known as the global ionosphere maps (GIMs) in the IONEX (ionosphere exchange) format. GIMs provide vertical TEC (VTEC) estimates (here we simply call it TEC) in terms of the spherical harmonics expansion up to degree and order 15 or in the grid domain with the spatial resolution of $2.5^{\circ }\times 5^{\circ }$ in latitude and longitude, respectively. Their temporal resolution is 15 min to 2 h. These products are available with a latency of less than 24 h and approximately 11 days in the rapid and final solution modes, respectively^52^. In this study, the rapid global TEC maps with 15 min time interval obtained from the UPC center^53^ are used for validating TEC estimates of TIE-GCM-I, globally.

### NRLMSISE-00 model

The Naval Research Laboratory’s Mass Spectrometer Incoherent Scatter Extension 2000 (NRLMSISE-00) model is an empirical model to simulate thermospheric variables^22^. The difference between NRLMSISE-00 and new version NRLMSISE2.0 can be found in the model outputs at low altitudes due to the consideration of new data below 100 km, so the impact of new model at the GRACE and Swarm orbits is negligible. The model uses various sources of data, see Fig. 2.9 in Kodikara (2019)^54^ including drag measurements and satellite-borne accelerometer data, incoherent scatter radar, mass spectrometer and pressure gauge over several decades (1961–1998)^22^. The model estimates depend on the altitude, geodetic latitude and longitude, local apparent solar time, solar flux for the previous day ($F_{10.7}$)^55^ and its 81-day average ($F_{10.7A}$), geomagnetic index (*Ap*)^56^ and 3205 model coefficients: *pavgm*, *pd*, *pdl*, *pdm*, *pma*, *ps*, *pt*, *ptl*, *ptm* and *sam*, which are adjusted during the model’s calibration period, and used to compute the number densities of He, O, N2, O2, Ar, H, N, anomalous oxygen and quantities such as TND, as well as neutral temperature.

To increase the efficiency of C/DA, the global sensitivity analysis (GSA) method^57^ is applied to identify key parameters with high impact on simulating TNDs in NRLMSISE-00. These parameters can be effectively calibrated by the accelerometer-derived TND estimates, see also Forootan et al. (2020)^47^. The covariances between the TND estimates and nineteen parameters of NRLMSISE-00 are shown in the Supplementary file (Section S4 online (Fig. S1)), which indicate the consistency between the GSA results from Forootan et al. (2020)^47^ and the empirical covariances between ensembles and model parameters. These results show that the first component of *ptm* and *pt* model coefficients, which are associated with estimating exospheric temperature, have the largest impact on estimating TNDs. To account for the sudden changes in solar and magnetic activities, which are not well reflected in the model coefficients, we replace the two constants of 150 and 4 in the model structure that are used to compute the index anomalies, i.e., $dF_{10.7A}=F_{10.7A}-150$ and $dAp=Ap-4$ are changed to $dF_{10.7A}=F_{10.7A}-C_{F_{10.7A}}$ and $dAp=Ap-C_{Ap}$. Therefore, these four variables (*ptm*(1), *pt*(1), $C_{F_{10.7A}}$ and $C_{Ap}$) are estimated within the C/DA procedure. The GRACE derived TND estimates during February 2015 are used as an example to test the performance of the C/DA approach.

### Thermosphere-ionosphere-electrodynamics general circulation model (TIE-GCM)

TIE-GCM is a coupled thermosphere-ionosphere model that uses a finite difference scheme to solve the nonlinear equations of conservation of mass, energy, and momentum for neutral and ion species^58,59^. This study is based on the TIE-GCM version 2.0 (released on March 21st, 2016). The horizontal resolution of this model is set to $5^{\circ }\times 5^{\circ }$, and the vertical resolution is two levels per scale height. The altitude of the model extends from approximately 97 km to 450–600 km depending on the solar activity^54^. In the TIE-GCM, the EUVAC (Extreme Ultraviolet Flux model for Aeronomic Calculations) empirical solar proxy model^60,61^ provides the solar irradiance inputs via the daily $F_{10.7}$ and its 81-day averaged ($F_{10.7A}$) time series. This model uses the *Kp* index^62^ instead of the *Ap* index for reflecting geomagnetic activity. Throughout this work, the Heelis model is been used to specify the high latitudes ion convection^63^. The Global Scale Wave Model (GSWM) provides the lower boundary condition, which is related to the atmospheric tides^64^.

To run TIE-GCM, one needs to introduce primary history files, which include the prognostic fields that are necessary to start the model. These files contain variables such as the neutral and ion temperature, neutral zonal and meridional wind, molecular and atomic Oxygen, Nitric Oxide, Helium, Argon, O$^{+}$ ion, electron temperature and density, O$_{2}^{+}$ ion, vertical motion, geopotential height and electric potential^59^. The differences between thermospheric constituents derived from TIE-GCM and C/DA-NRLMSISE-00 is discussed in the Supplementary file (Section S5 online). The density of atomic oxygen (O), molecular oxygen ($\mathrm O_{2}$), helium (He), and neutral temperature (TN) from the C/DA-NRLMSISE-00 will replace the primary history files of TIE-GCM (thus named as TIE-GCM-I) to test their impact on the forecasts of TEC and electron density variables.

## Methods

### Calibration and data assimilation (C/DA) framework to form C/DA-NRLMSISE-00

In this study, the simultaneous C/DA^46,47^ technique is applied to integrate accelerometer-derived TND measurements into NRLMSISE-00. The C/DA approach contains a model-state equation, where the model derived integral of all neutral mass estimates, i.e., TNDs, and four model parameters are the unknowns. These are computed simultaneously through a sequential ensemble Kalman filter (EnKF)^65^ procedure. First, let us assume that our original model, i.e., NRLMSISE-00^22^, is mathematically represented as:

1 $$\begin{aligned} \text {Original model, i.e., NRLMSISE-00}: F({\Theta })=F({\Theta _P},{\Theta _R},{\Theta _I}), \end{aligned}$$

where ${\Theta }$ is a vector of model parameters and input values that enable the model to be run and simulate TND, individual mass densities, and thermospheric temperature at arbitrary locations and time steps. In Eq. (1), we consider that ${\Theta }$ contains ${{\Theta }_P}_{4\times 1}$ that are the four key parameters from the global sensitivity analysis (discussed in the Supplementary file, Section S6 online) and will be updated during the C/DA procedure. Those parameters that remain unchanged during C/DA is shown by ${{\Theta }_R}$. Finally, ${{\Theta }_I}$ indicates the input variables such as the values of solar and geomagnetic indices, location (longitude, latitude, and height), and time.

During the C/DA procedure, accelerometer-derived TNDs are used as observations at the time step to correct the TND states of NRLMSISE-00 and to calibrate its key (here 4) parameters through minimizing the following cost function:

2 $$\begin{aligned} J(\mathbf{X })= \frac{1}{2}[\mathbf{X }-\bar{\mathbf{X }}^{b}]^{T} (\mathbf{P }^{b})^{-1} [\mathbf{X }-\bar{\mathbf{X }}^{b}] + \frac{1}{2}[\mathbf{H }\mathbf{X }^{b}-\mathbf{Y }]^{T} \mathbf{R }^{-1} (\mathbf{H }\mathbf{X }^{b}-\mathbf{Y }), \end{aligned}$$

where *N* is the ensemble member ($N=75$), and $\mathbf{X }_{k}^{b}=[\mathbf{x }_{k}^{b(1)}, \cdots ,\mathbf{x }_{k}^{b(N)}]$ is the ensemble of background state (i.e., $\mathbf{x }^{b(i)} \in \mathfrak {R}^{n}$) and is composed of two parts: the ensemble of model state and model parameters. $\bar{\mathbf{X }}^{b}$ is the ensemble mean vector and $\mathbf{P }_{k}^{b}$ is the background error covariance. Ensembles of observations are stored in $\text {Y}_{k}=[\mathbf{y }^{1}, \cdots ,\mathbf{y }^{N}]$ (i.e., $\mathbf{y }^{i} \in \mathfrak {R}^{m}$), which are perturbed by $\mathbf{R }_{k}$, which is the covariance matrix of observations. Assuming that the measurements of TNDs to be independent, the magnitude of biases in Fig. 1b, transformed to the satellites’ altitudes, is considered as uncertainty.

The analysis state estimates ($\mathbf{X }^{a}$) are obtained while taking into account the measurements and the cross-correlations of model states and parameters as:

3 $$\begin{aligned} \mathbf{X }^{a}=\mathbf{X }^{b}+\mathbf{K }(\mathbf{Y }-\mathbf{H } \mathbf{X }^{b}), \end{aligned}$$

where $\mathbf{K }$ is the Kalman gain, and the design matrix ${\mathbf{H }}: \mathfrak {R}^{n} \rightarrow \mathfrak {R}^{m}$ connects the model states and parameters to the observations. Equation (3) was evaluated at time *k* to obtain the ensemble of analysis estimate $\mathbf{X }^{a}$. The state can then be used for forecasting $\mathbf{X }^{b}$ of the next step and the C/AD process continues until the observations are available. The C/DA procedure of this study is performed using 3 h of GRACE-TNDs. The last set of key parameters derived from the last ensemble mean (in Eq. (3)) are considered as the updated key parameters shown by ${\hat{\Theta }}_P$. These parameters then replace the default values of the original NRLMSISE-00 model (Eq. (1)) to form the C/DA-NRLMSISE-00 model as:

4 $$\begin{aligned} \text {C/DA-NRLMSISE-00 model, i.e., }: \text {F}({{\hat{\Theta }}_P}, {\Theta _R},{\Theta _I}), \end{aligned}$$

C/DA-NRLMSISE-00 can be applied to estimate TNDs globally (i.e., even the regions that are not covered by GRACE orbits) and for forecasting the global map of TNDs, individual mass densities and thermospheric temperature of the next 1 h to 21 h (see the next section). The mathematical details of C/DA are described in the Supplementary file (Section S6 online).

### Statistical measures to examine the quality of model simulations

To evaluate the performance of models, the following statistical metrics are applied.

- The Root-Mean-Squares of Error (RMSE) is applied as a scale-dependent measure to extract how well these models agree with the measurements as:
  5 $$\begin{aligned} \text {RMSE}=\sqrt{\frac{\sum _{i=1}^{n}(\text {Obs}-\text {Model})^{2}}{n}}, \end{aligned}$$
  where $\text {Obs}$ and $\text {Model}$ denote observation and model estimates, receptively and *n* is the number of observations.
- The reduction of RMSE is interpreted as ‘improvement’, which is computed as: where $\text {RMSE}_1$ is the differences between the original model (e.g., NRLMSISE-00 or TIE-GCM) and observations (e.g., neutral densities from GRACE, Swarm-A /-B and -C, and electron density profiles from RO), and $\text {RMSE}_2$ is determined from differences between C/DA-NRLMSISE-00 or TIE-GCM-I models and observations.
  6 $$\begin{aligned} \text {Improvement}=100\times \frac{\text {RMSE}_1-\text {RMSE}_2}{\text {RMSE}_1}, \end{aligned}$$
  The Average Absolute Percentage Deviations (AAPD) is used as a scale-independent measure, i.e.,
  7 $$\begin{aligned} \text {AAPD}=\frac{\sum _{i=1}^{n}(100\times |\frac{\text {Obs}_{i}-\text {Model}_{i}}{\text {Obs}_{i}}|)}{n}, \end{aligned}$$
  where, the minimum (maximum) values for AAPD indicate that the model provides on average best (worst) performance in prediction.
- The Normalized Root Mean Square Error (NRMSE) is defined by comparing the models against the observations to represent the fraction of data-variance that can be explained by these models. The computation follows:
  8 $$\begin{aligned} \text {NRMSE}=1-\frac{\sqrt{(\text {Obs}-\text {Model})^{2}}}{\sqrt{(\text {Obs}-\bar{\text {Obs}}))^{2}}}, \end{aligned}$$
  where $\bar{\text {Obs}}$ is defined as the mean of observations. In contrast to AAPD, the minimum (maximum) values for NRMSE indicate that the model provides on average worst (best) performance in estimating the variable of interest.
- The expression of difference between model and observation in percentage is computed based on the ’Relative Error (RE)’ as:
  9 $$\begin{aligned} \text {RE}=100\times \frac{|\text {Obs}-\text {Model}|}{\text {Obs}}, \end{aligned}$$
  where $|. |$ computes the absolute values.

### Setup of the C/DA-NRLMSISE-00 model

**Figure 2:**
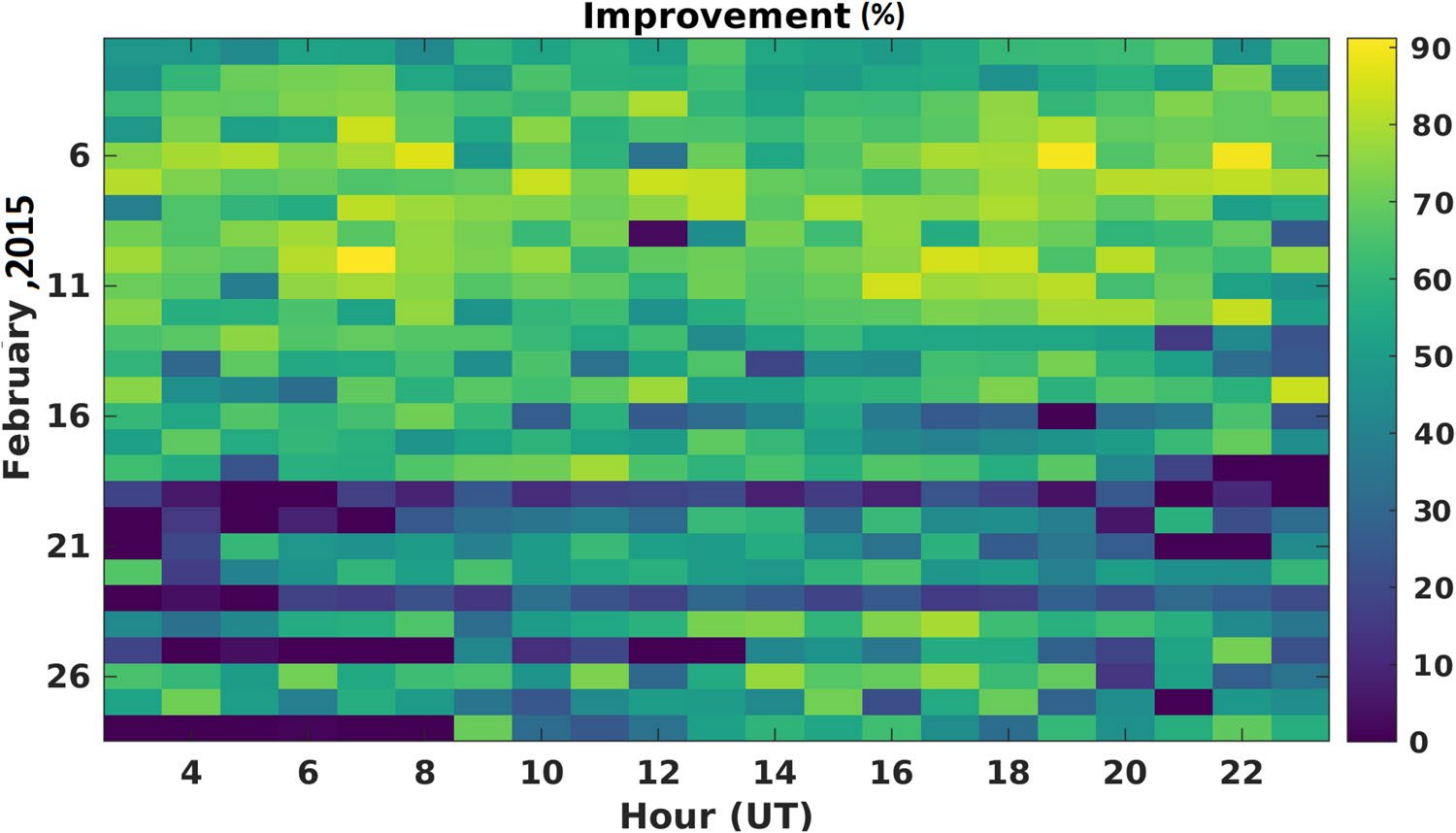
An overview of the improvements (from Eq. (6)) in forecasting TNDs during February 2015. The results are estimated by running the original NRLMSISE-00 and C/DA-NRLMSISE-00. The C/DA is processed using GRACE-TNDs as observations, then the 3-hourly calibrated parameters are used to form the C/DA-NRLMSISE-00 model and forecasting TNDs along Swarm-C during different times of the month. The quality of forecasts is found to be 50.62$\%$ improved, on average. The plot was made in MATLAB (version R2021a, https://www.mathworks.com/).

The specifics of the C/DA configuration and parameter settings used in this study are as follows: the ensemble size is selected to be 75. The assimilation window size is determined experimentally by changing its size from 1 to 5 h. In each experiment, the calibrated parameters were used to predict the TNDs of the next hours. The minimum RMSE (Eq. 5), against Swarm-A, -B, and -C TNDs, was found to be related to the window size of 3 h. To define an optimal period of the forecast, we implemented different prediction periods from next 1 h to the next 21 h. This means that, for example, the TNDs of 00:00:00 UT to 02:59:30 h UT are used as observations, then the C/DA-NRLMSISE-00 predicts the neutral density values for the next 21 h from 03:00:00 UT to 23:59:30 UT. Figure 2 indicates the improvements in the RMSE values of forecasts for all the 21 h of each day during February 2015. The results indicate that the quality of forecast are generally high ($50.62\%$ on average) in most of the days. During days with low (e.g., February 20th, 2015) or sudden changes in the geomagnetic index (e.g., February 24th, 2015), the quality is slightly less (32.34 and $13.46\%$ on average), which can be related to the uncertainties of F10.7 and Ap indices in the forecasting mode. But overall, the C/DA-NRLMSISE-00 is found to be better than the original NRLMSISE-00 in simulating TNDs.

The initial ensembles of C/DA are generated by perturbing the key parameters (i.e, *ptm*(1),*pt*(1),$C_{F.107}$ and $C_{Ap}$) for each ensemble member *N* and the parameters are sampled from a normal distribution. The mean values for the key parameters *ptm*(1), *pt*(1), $C_{F.107}$ and $C_{Ap}$ are 1041.3, 0.9865, 150 and 4, respectively, corresponding to the default values in the original NRLMSISE-00 model. The variances of the perturbation for *ptm*(1), *pt*(1), $C_{F.107}$ and $C_{Ap}$ are selected to be 10.41, 0.0098, 2 and 1, respectively (i.e. around 10% of their default values). During the 3 h of C/DA window, four parameters of ${\hat{\Theta }}_P$ are estimated, then they are used in Eq. (4) for forecasting thermospheric variables.

## Results and discussion

**Figure 3:**
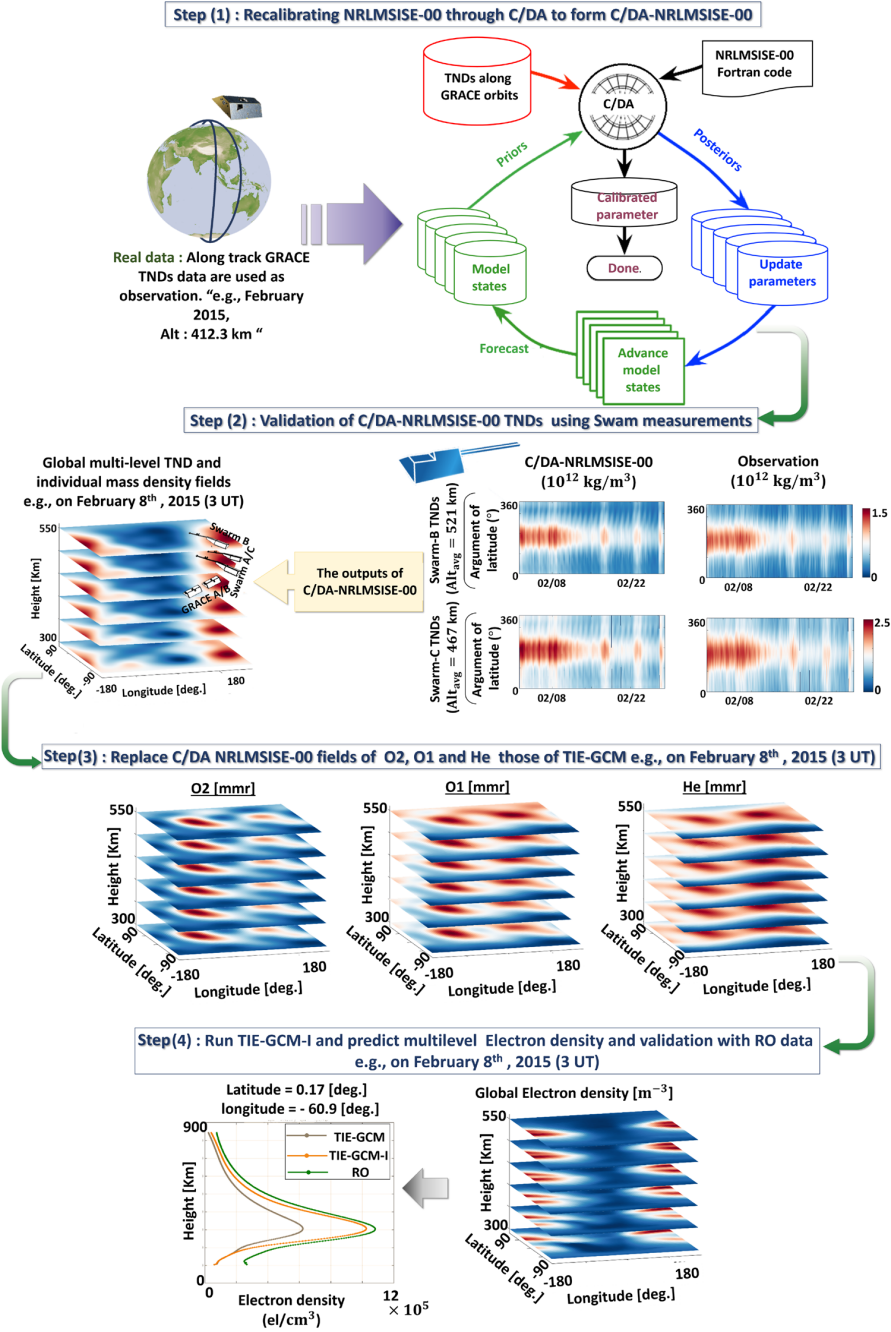
An overview of the proposed C/DA procedure and validation experiments. The procedure is divided into four steps: (1) Applying the C/DA on the NRLMSISE-00 model to form C/DA-NRLMSISE-00, (2) Validating TNDs against Swarm measurements, (3) Improving the primary history files of TIE-GCM using the forecasting values provided by C/DA-NRLMSISE-00, and (4) validating the ionospheric variables using GNSS and RO measurements. The plots were generated using MATLAB (version R2021a, https://www.mathworks.com/) and PowerPoint (2016, https://www.microsoft.com/) was used for producing the figure.

An overview of the work-flow of this study to apply C/DA on NRLMSISE-00 and testing its performance in forecasting thermospheric and ionospheric variables is presented in Fig. 3. The procedure is started by (1) using the GRACE-TND observations to re-calibrate the four key parameters of NRLMSISE-00 (i.e, *ptm*(1),*pt*(1),$C_{F.107}$ and $C_{Ap}$) through C/DA. In (2), the TND values during the 1-h to 21-h forecast mode are compared with the independent measurements derived from Swarm. In (3), some thermospheric constituents derived from the C/DA-NRLMSISE-00 model are used to replace the primary history files of TIE-GCM to test whether the interactions between thermosphere and ionosphere can be improved. Another validation step is performed in (4), the original TIE-GCM and TIE-GCM-I (with improved primary history files) is compared with ionospheric observations such as TEC maps^66^ and RO profiles^67^.

### Along-track validation of TNDs from C/DA-NRLMSISE-00 during the analysis phase

**Figure 4:**
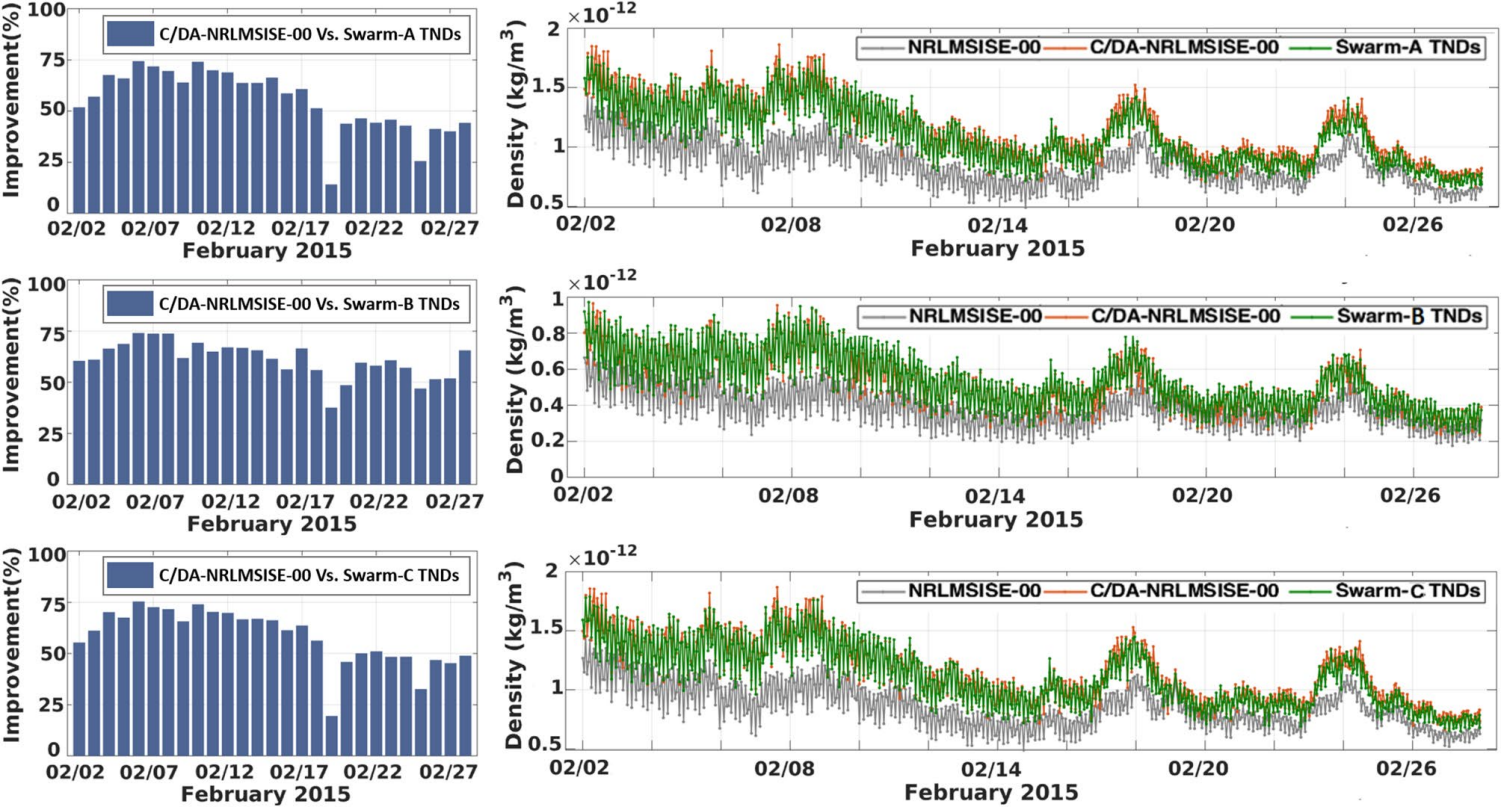
A comparison of the estimated improvements (Eq. (6)) and the time series of TNDs derived from the original NRLMSISE-00 and C/DA-NRLMSISE-00 models along with Swarm TNDs during February 2015. The C/DA is processed using GRACE-TNDs as observations, then the C/DA-NRLMSISE-00 densities are evaluated along the Swarm-A, -B and -C orbits. The plots were made in MATLAB (version R2021a, https://www.mathworks.com/).

The focus of this study is on forecasting TNDs based on C/DA. However, it is essential to validate whether this method can improve NRLMSISE-00 during the data assimilation window (analysis phase). The validation can be carried out using the data that were not used in the C/DA procedure. Thus, here, we use the GRACE-TNDs as observation of the C/DA, and the TNDs along the Swarm-A/-B and -C orbits are used for validation. Figure 4(left) presents the improvements in terms of RMSEs between the TNDs of NRLMSISE-00 and C/DA-NRLMSISE-00. In addition, Fig. 4(right) shows the time series of TNDs (during February 2015) used for these estimations. Based on the statistical results shown in Table 1, after implementing the C/DA, the overall RMSE during the entire month is reduced by 53, 59, and 56%, respectively, along Swarm-A, Swarm-B, and Swarm-C orbits compared to the original model.

These numerical results illustrate the ability of C/DA-NRLMSISE-00 in estimating TNDs with high accuracy compared to the original model. Please note that during these experiments only calibrated parameters derived from the C/DA procedure are used to run the model in the analysis and forecasting phases. We do not present the updated TNDs that are co-estimated with the four parameters, because those values are well fitted to GRACE measurements during the analysis phase due to the least squares optimization. Therefore, from the results presented in this paper, the fit of the estimates during the analysis phase is not 100%. For instance the fitting parameter between model and observation is increased (in terms of NRMSE) from -0.09 , 0.16 and -0.07 to 0.48, 0.64 and 0.53 along the Swarm-A, Swarm-B, and Swarm-C orbits, respectively. Nevertheless, the fitted estimates validated with the data from the Swarm mission is presented in Fig. 4 and Table 1. We also found a drop in the estimated improvements around February 19th, 2015 but this does not correspond to the lack of the skill of the two models. This is caused by the small differences between the absolute values of models and measurements (see Fig. 4(right)). A statistical T-test is performed between the observation and TNDs from NRLMSISE-00 and C/DA-NRLMSISE-00 along the Swarm-A, Swarm-B, and Swarm-C orbits. The significance level was set at 90%. The test decision indicates that the changes in the performance of C/DA is statistically significant. In the light of these positive results, we further assess C/DA-NRLMSISE-00 during the forecast phase.

**Table 1:** A summary of the statistical measures derived during the analysis mode between the original NRLMSISE-00 and C/DA-NRLMSISE-00 models. Here GRACE-TNDs are used as observations within the C/DA procedure, and the results are evaluated with those of the Swarm mission during February 2015.

Mission	Model					
	RMSE (kg/$\mathrm{m}^3$)		NRMSE		AAPD (%)	
	NRLMSISE-00	C/DA-NRLMSISE-00	NRLMSISE-00	C/DA-NRLMSISE-00	NRLMSISE-00	C/DA-NRLMSISE-00
Swarm A	$2.76\times 10^{-13}$	$1.16\times 10^{-13}$	− 0.09	0.48	22.23	9.17
Swarm B	$1.77\times 10^{-13}$	$7.05\times 10^{-14}$	0.16	0.64	28.61	12.29
Swarm C	$2.86\times 10^{-13}$	$1.14\times 10^{-13}$	− 0.07	0.52	22.76	8.93

### Forecasting of C/DA-NRLMSISE-00 TNDs along the daily tracks of GRACE and Swarm

**Figure 5:**
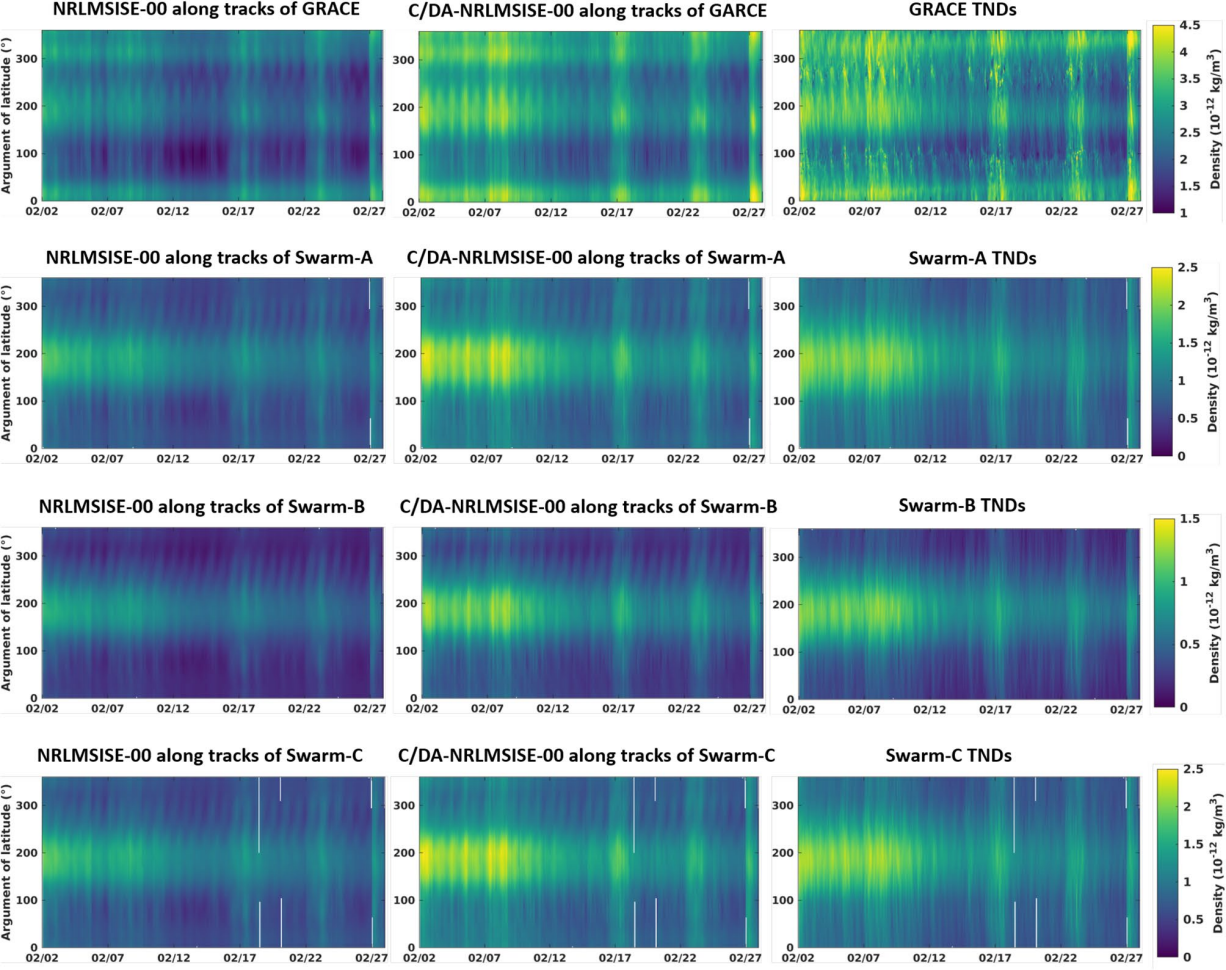
Maps of TND estimates retrieved from NRLMSISE-00 and C/DA-NRLMSISE-00 in the forecasting mode, as well as TND estimates derived along track of GRACE, Swarm-A, -B, and -C. The C/DA-NRLMSISE-00 model is implemented based on the description in the ‘Setup of the C/DA-NRLMSISE-00 Model’ section. The plots were generated using MATLAB (version R2021a, https://www.mathworks.com/).

In this section, the estimated model parameters for each data assimilation window (i.e., 3 h) are applied to forecast TNDs for the next hour. Figure 5 presents the forecast of TND maps derived from the original NRLMSISE-00, C/DA-NRLMSISE-00 and those of GRACE and Swarm as a function of time and the argument of latitude (the angle along the orbital path from the ascending node to the spacecraft’s position in the direction of the spacecraft’s motion) during 27 days (February 2nd–28th, 2015). A visual comparison indicated that the TNDs of C/DA-NRLMSISE-00 (in the forecast phase) are much closer to the observations. Higher spatial correlation of 98.28, 98.25, 97.53, and $98.25\%$ are found between C/DA-NRLMSISE-00 and GRACE, Swarm-A, -B, and -C compared to those of the original NRLMSISE-00, i.e., 88.34, 90.76, 92.01, and $90.75\%$.

**Figure 6:**
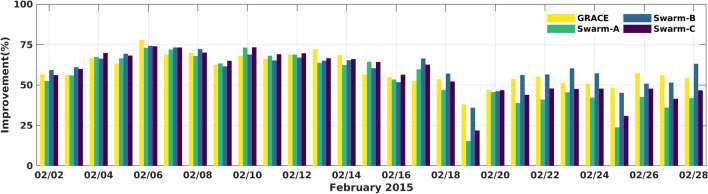
An overview of improvements in percent, which is used to assess the performance of the C/DA based on GRACE-TNDs as observation in the C/DA framework during February 2015. The figure was generated using MATLAB (version R2021a, https://www.mathworks.com/).

An average of the daily assessment during the forecast mode (based on Eq. (4)) is presented in Fig. 6, where an average improvement of $62.98\%$ is obtained during February 2nd–17th, 2015 and an average $39.54\%$ during February 18th–28th, 2015. The small improvement value during the second period corresponds to the considerable geomagnetic variably, reflected in the *Ap* values (see Fig. 1) that negatively affects the optimization using a short-length C/DA window. Nevertheless, these improvements show that C/DA is very effective during days with medium and high solar activity (see Fig. 1, panel (c)), where the original NRLMSISE-00 shows considerable uncertainties^68^. The monthly average of statistical measures during the forecasting mode is provided in Table 2.

**Table 2:** A summary of statistical measures between the original and C/DA-NRLMSISE-00 compared to the TNDs derived from GRACE and Swarm measurements during February 2015 in the forecasting mode.

Mission	Model					
	RMSE(kg/$\mathrm{m}^3$)		NRMSE		AAPD (%)	
	NRLMSISE-00	C/DA-NRLMSISE-00	NRLMSISE-00	C/DA-NRLMSISE-00	NRLMSISE-00	C/DA-NRLMSISE-00
GRACE	$7.10\times 10^{-13}$	$2.79\times 10^{-13}$	− 0.54	0.39	22.11	7.36
Swarm A	$2.68\times 10^{-13}$	$1.09\times 10^{-13}$	− 0.09	0.49	21.95	8.89
Swarm B	$1.73\times 10^{-13}$	$6.60\times 10^{-14}$	0.16	0.66	28.40	11.75
Swarm C	$2.78\times 10^{-13}$	$1.07\times 10^{-13}$	− 0.06	0.54	22.48	8.62

### Multi-level variations of the thermospheric temperature and neutral density

**Figure 7:**
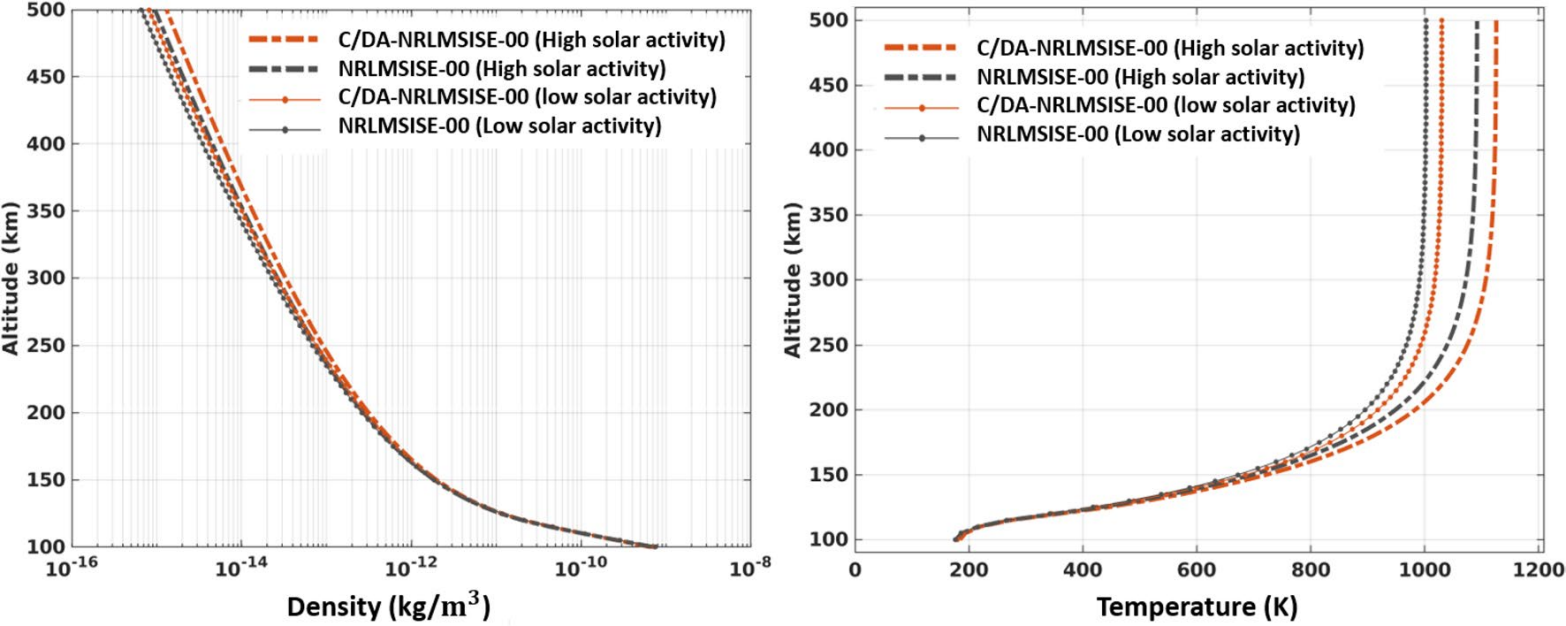
Temperature and density profiles during low solar activity (February 26th, 2015 at 12:00 UT, $F_{10.7} = 110$, gray) and high solar activity (February 8th, 2015 at 12:00 UT, $F_{10.7} = 153$, red) conditions. The solid and dashed lines are the corresponding NRLMSISE-00 and C/DA model, respectively. The vertical profile is estimated at latitude $0^{\circ }$ and longitude $0^{\circ }$. The plots were generated using MATLAB (version R2021a, https://www.mathworks.com/).

The four calibrated parameters do not only affect the simulation of thermospheric variables at the altitude of the assimilated observations (here GRACE TND data), their impacts can also be sensed on other altitudes mainly because of the relationship between the two parameters (*ptm*(1) and *pt*(1)) and the calculation of thermospheric temperature. To illustrate the changes, temperature and density profiles of 100-500 km are presented in Fig. 7, where they correspond to two days with low and high solar activity on February 26th and 8th, 2015 at 12:00 UT. These profiles are shown by the grey and red lines, respectively, and they are associated with the latitude $0^{\circ }$ and longitude $0^{\circ }$. These results indicate that by applying C/DA both profiles are modified, whereas the temperature during high (low) solar activity was on average increased by 34.34 (43.26) degree covering the altitude of 200 to 500 km. This is equivalent with $7.52\times 10^{-12}$ ($8.50\times 10^{-12}$) changes in density estimates, respectively. It is worth mentioning that the changes in thermosphere are more complex during storms. Especially, variations due to winds become more pronounces and the barometric processes get violated, see e.g., Lie et al.^69^. Therefore, adding wind and temperature measurements to the C/DA might further improve the results during these events. In this study, our assumption is that the density changes derived from accelerometer measurements are de-coupled from the wind component because this is the methodology that is applied for estimating accelerometer-derived TNDs (see Doornbos^70^). Further investigations will be performed in future to apply C/DA on the coupled neutral mass density and wind models.

**Figure 8:**
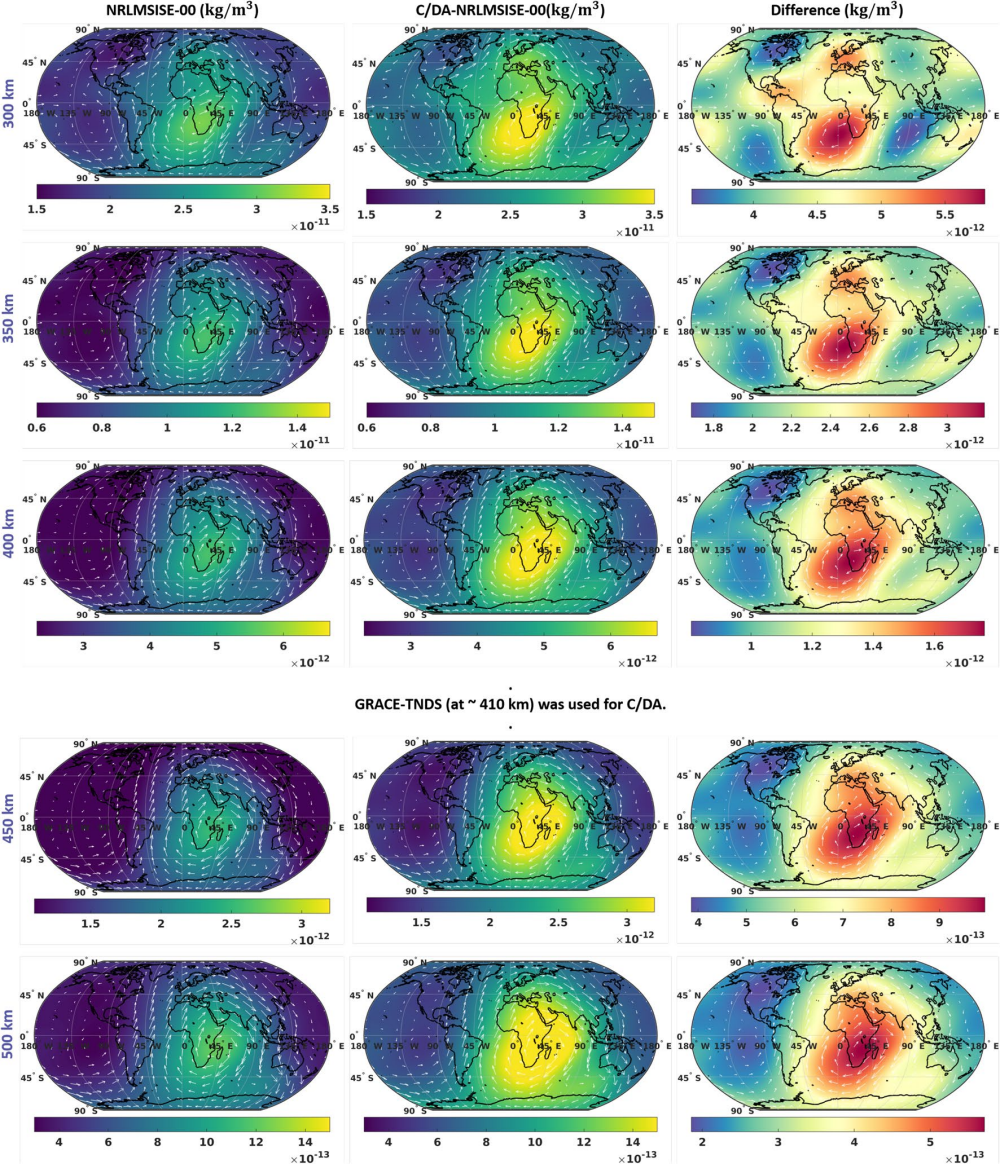
(left) Global maps of thermosphere neutral density (TND) from the original NRLMSISE-00, (middle) the forecasts of C/DA-NRLMSISE-00 with 1 h lead time, and (right) their difference at 12h UT on February 8th, 2015 for five different altitudes. The contour lines present the gradient vectors of density. These plots were generated using MATLAB (version R2021a, https://www.mathworks.com/).

In Fig. 8, global maps of TND at different altitudes of 300, 350, 400, 450 and 500 km at the universal time 12:00 are shown. The maps shown from left to right correspond to those from NRLMSISE-00, C/DA-NRLMSISE-00, and the difference between them during February 8th, 2015 (a day with high solar activity, $F_{10.7}=153$). The maps on the right columns indicate that C/DA changes the magnitude of TNDs, not only affect the simulation at the GRACE altitude of 410 km ($30.7\%$ on average) but also changes the TNDs of other altitudes especially around the longitude $0^{\circ }$ associated with the local noon, (i.e., 300 km: $22.86\%$, 350 km: $26.47\%$, 400 km: $29.96\%$, 450 km: $33.54\%$, and 500 km: $37.11\%$). It should be considered that the C/DA procedure is implemented using GRACE-TNDs that belong to the medium altitude (e.g., 400-500 km), thus, it might be difficult to interpret the TNDs of much higher or lower altitudes, or where the composition of neutral mass density considerably changes. This is due to the fact that the temperature and density profiles (Fig. 7), as well as the global TND maps on different altitudes (Fig. 8) are computed based on the vertical assumption of NRLMSISE-00 and the C/DA techniques does not violate the physical concept that is implemented in the model.

### Comparing the equatorial thermospheric neutral density anomalies

**Figure 9:**
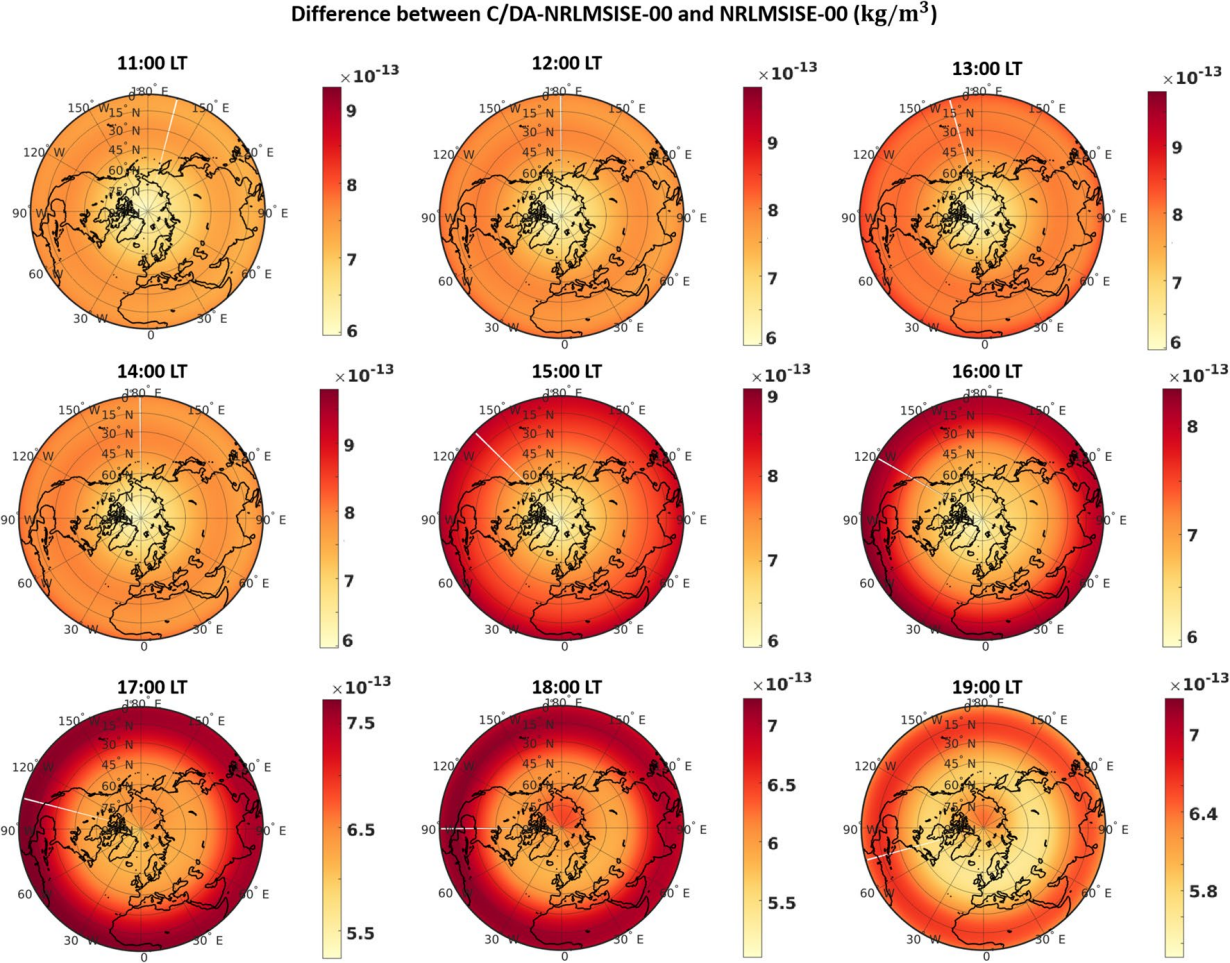
Thermosphere neutral density (TND) maps at the altitude of 450 km derived from the original NRLMSISE-00 (plots on left) and C/DA-NRLMSISE-00 (plots on middle) during 12 to 18 h local time (LT) on February 8th, 2015. The plots on the right correspond to the relative error (RE) maps that illustrate the impact of C/DA on representing the equatorial thermosphere anomaly (ETA). MATLAB (version R2021a, https://www.mathworks.com/) was used to generate these plots.

In this section, we explore the impact of C/DA on representing the characteristics of the equatorial thermosphere anomaly (ETA)^71^. Analogous to the equatorial ionosphere anomaly (EIA)^72^, ETA is formed around the local noon. Various mechanisms are named as drivers of ETA namely heat transport due to zonal winds, chemical heating, and field-aligned ion drag^73^. ETA is then represented as a function of local time, season, geomagnetic and solar activity index. To illustrate the effect of C/DA on representing ETA, the differences between are computed during different local times. The differences polar map for the altitude of 450 km are depicted in Fig. 9 on February 8th, 2015 with relatively high solar activity. The patterns of difference between TNDs from 11:00 to 19:00 LT indicate that the peak values locate around the ETA. In addition, the ETA features become more prominent at 15:00 LT due to its daytime phenomenon and tight coupling with the EIA, which disappear rapidly after sunset. These results indicate that the C/DA increases the magnitude of TND changes due to ETA up to about 20-30$\%$ . Therefore, we conclude that the under-estimation of TNDs from the original model can be eliminated by tuning it through the C/DA and using the accelerometer derived TND as observations.

### Exploring the spatial and temporal impacts of C/DA through the principal component analysis (PCA)

**Figure 10:**
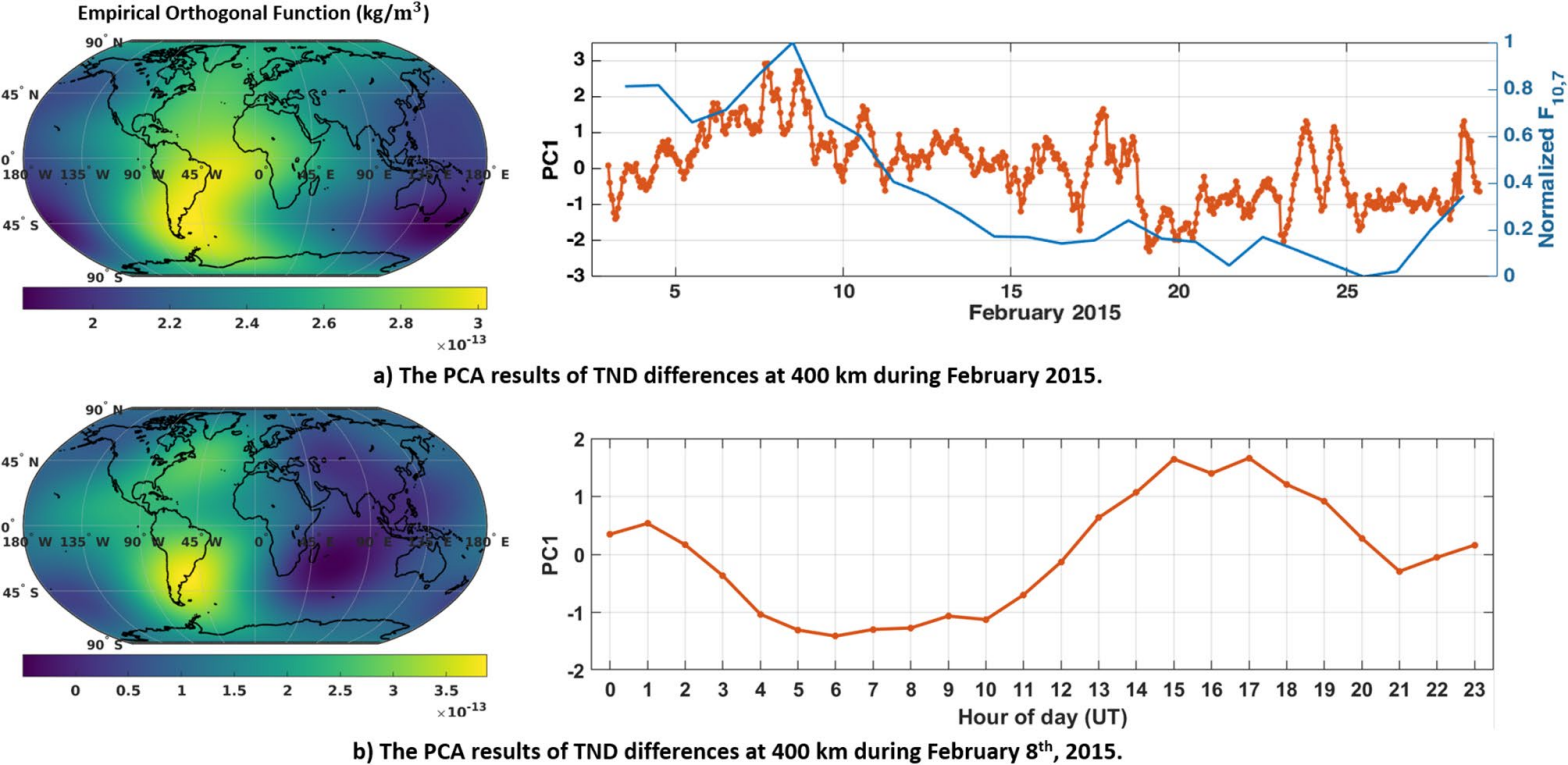
PCA of the TND differences between NRLMSISE-00 and C/DA-NRLMSISE-00 at 400 km during February 2015. The left plots are anomaly maps (EOFs) in terms of kg/m$^{3}$, which can be multiplied by the unit less time series (PCs) on the right to derive orthogonal modes. The first mode of monthly differences (on top) represents $75.55\%$ of the total variance of TND differences and the first mode of daily differences (on bottom) mode indicates $52.34\%$ of the variance. These plots were generated using MATLAB (version R2021a, https://www.mathworks.com/).

To investigate the dominant spatial and temporal changes caused by applying the C/DA method, the principal component analysis (PCA)^34,74^ method is applied on the hourly differences between NRLMSISE-00 and C/DA-NRLMSISE-00 during one day (i.e., February 8th, 2015) and entire February 2015. The mathematical details of PCA are described in the Supplementary file (Section S7 online). The global differences at the altitude of 400 km during February 2015 are presented in Fig. 10. The spatial patterns known as empirical orthogonal functions (EOFs that are anomaly maps in terms of density kg/m$^{3}$) and their associated uncorrelated temporal patterns (principal components, or PCs that are unit-less) represent the orthogonal modes and are plotted beside each other.

The first dominant mode corresponds to $75.55\%$ and $52.34\%$ of the total variance of monthly and daily TND differences, respectively. The first mode of monthly differences in Fig. 10 (the global anomaly map and its temporal PC on top) indicates that the average difference in terms of TNDs is found to be $7.13\times 10^{-13} \, \mathrm{kg}/\mathrm{m}^{3}$ (in the range of $-1.06\times 10^{-13} - 2.20\times 10^{-12} \,\mathrm{kg}/\mathrm{m}^{3}$). The results also show that the magnitude of differences during February 1st–10th is higher than the rest. PC1 is found to be correlated with the solar activity index with the correlation coefficient of $50.04\%$ (see Fig. 1) shows the standardized solar index along-side of PC1. This confirms the previous results of e.g., Forootan et al.^32^ who reported that empirical models do not reflect recent neutral mass density changes caused by solar activity. The first mode of daily TND differences Fig. 10 (the global anomaly map and its temporal PC on the bottom of figure) indicates that the magnitude of differences reach up to $10^{-12}\,\mathrm{kg}/\mathrm{m}^{3}$, and it is dominated by the diurnal frequency. The impact of these differences on the drag acceleration estimation is equivalent with 17.79, 33.42, 50.87 and $68.16 \%$ on a satellite at 300, 400, 500 and 600 km altitude thus it cannot be ignored in the precise orbit determination applications. It should be mentioned here that the atmospheric drag depends on the cross-sectional area perpendicular to the velocity vector of satellite (*A*), mass of the satellite (*m*), drag coefficient ($C_{D}$), density ($\rho$), the norm of the relative velocity vector (${v}_{rel}^{2}$), and the direction of the velocity relative to the co-rotating atmosphere (${\hat{v}}_{rel}$). The values presented above are determined for spherical satellites (${a}_{drag}=\frac{1}{2}\frac{A}{m}{C_{D}}\;\rho \;{v}_{rel}^{2}{\hat{v}}_{rel}$,) with the area of $1.00135\,\mathrm{m}^{2}$, mass of 487.2 kg and velocity of $7600 \mathrm{m}\,\mathrm{s}^{-1}$ on average.

### Assessing the impact of updating TND estimates on forecasting ionospheric parameters

The main objective of this section is to demonstrate the impact of the thermospheric constituents from C/DA-NRLMSISE-00 on the forecasts of electron density. For this, the physics-based TIE-GCM model is chosen to simulate ionospheric variables. For this experiment the atomic oxygen (O), molecular oxygen ($\mathrm O_{2}$), helium (He), and neutral temperature (TN) from the outputs of the C/DA-NRLMSISE-00 model are used as the primary history files of TIE-GCM. This is done by converting the species abundances from C/DA-NRLMSISE-00 to the mass mixing ratio $\gamma$ with the estimation of conversion factor and this assumption $\gamma _{N_{2}} = 1-(\gamma _{O} + \gamma _{O_{2}} + \gamma _{He})$^59^, where $\gamma _{N_{2}}$, $\gamma _{O}$, $\gamma _{O_{2}}$, and $\gamma _{He}$ represent mass mixing ratios of molecular nitrogen, atomic oxygen, molecular oxygen, and helium, respectively. The conversion factor *C*, which relates the number of densities to the mass mixing ratio, is assumed to be $C=\frac{Pt_{i} \times \bar{m}}{B \times Tn}$, where $Pt_{i}$ represents the pressure of each midpoint level *i*, and ${\bar{m}}$ is the mean molecular mass (g/mol) estimated as ${\bar{m}}=\frac{1}{\frac{\gamma _{O2}}{32}+\frac{\gamma _{O}}{16}+\frac{\gamma _{He}}{4}+\frac{\gamma _{N2}}{28}}$, *B* is the Boltzman constant $B = 1.3805\times 10^{-16}$, and *Tn* is the neutral temperature (K). Finally, the number of density $cm^{-3}$ from C/DA-NRLMSISE-00 ($\rho _{C/DA-NRLMSISE-00}$) is converted to the mass mixing ratio (*mmr*) as $\rho _{mmr}=\frac{w \times \rho _{C/DA-NRLMSISE-00}}{C}$, where *w* is the molecular weight of each species (i.e., $w_{O2}=32$, $w_{O}=16$, $w_{He}=4$, and $w_{N2}=28$).

To form TIE-GCM-I, which is the same as the original TIE-GCM with the improved primary history files based on the C/DA-NRLMSISE-00, and forecasting ionospheric variables the following steps are taken: (1) $\gamma _{O2}$, $\gamma _{O}$, $\gamma _{He}$ and $\gamma _{N2}$ and neutral temperature are estimated through the C/DA-NRLMSISE-00 in the forecasting mode; (2) the estimated parameters are converted to *mmr* and used to replace the default values in the primary history files; and (3) TIE-GCM-I is run for 1 h with recording the model output every 5 min. These three steps are repeated for 24 h of each day.

We chose February 8th, 2015 with high solar activity to test the results. The standalone TIE-GCM and TIE-GCM-I are compared with TEC from IGS products, and the RO-derived electron densities. Note that the model has an upper boundary of around 500-800 km altitude, while TEC estimates of IONEX represent $\sim 20,200$ km altitude. To reduce this inconsistency, the TEC estimates above the upper boundary of models are added using the simulation of the NeQuick ionosphere model^75^.

**Figure 11:**
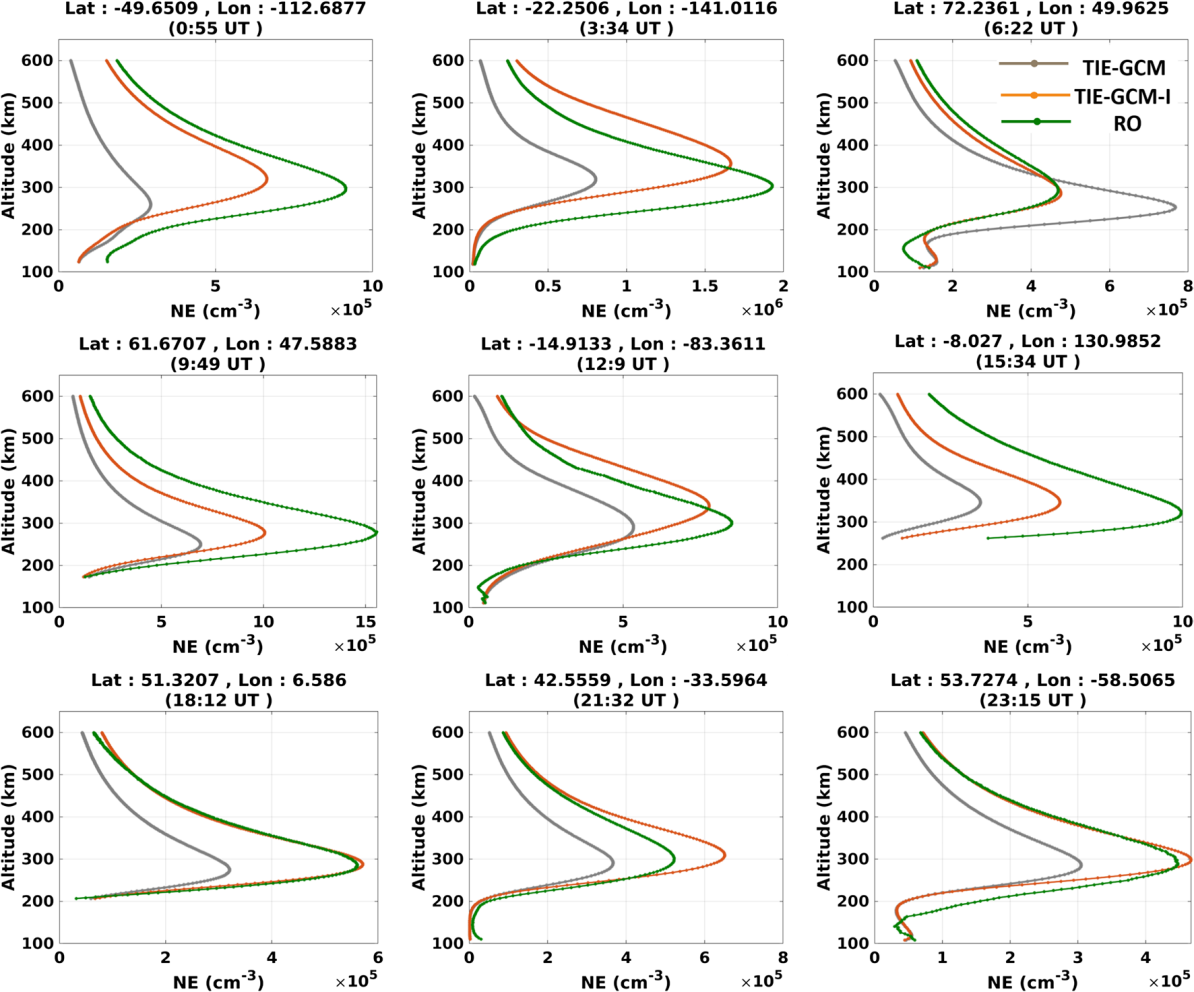
The altitude-dependent profiles of *Ne* retrieved from the COSMIC radio occultation data, as well as the original TIE-GCM, and TIEGCM-I during 24 h of February 8th, 2015. The plots were generated using MATLAB (version R2021a, https://www.mathworks.com/).

In Fig. 11, we present nine vertical profiles of the electron density from models and RO during 24 h that correspond to high, mid, and low latitudes. To compare the results in terms of electron density, the Bi-linear and cubic spline interpolation scheme^76^ was used to map the electron densities of TIE-GCM and TIE-GCM-I along the RO observations. Through the Comparisons with 300 COSMIC profiles during February 8th, 2015, we found the mean fitting coefficients (i.e., NRMSE based on the Eq. (8)) and the correlation coefficient to be 0.15 and $84.4\%$ between TIE-GCM-I and RO profiles, respectively. These values show a better performance since those between TIE-GCM and RO was found to be $-0.39$ and $74.7\%$. Considering the results of the entire month, the TIE-GCM-I considerably reduces the RMSE between the electron density forecasts and RO observations within the range of $0.2-90.6\%$ (on average $30.92\%$).

**Figure 12:**
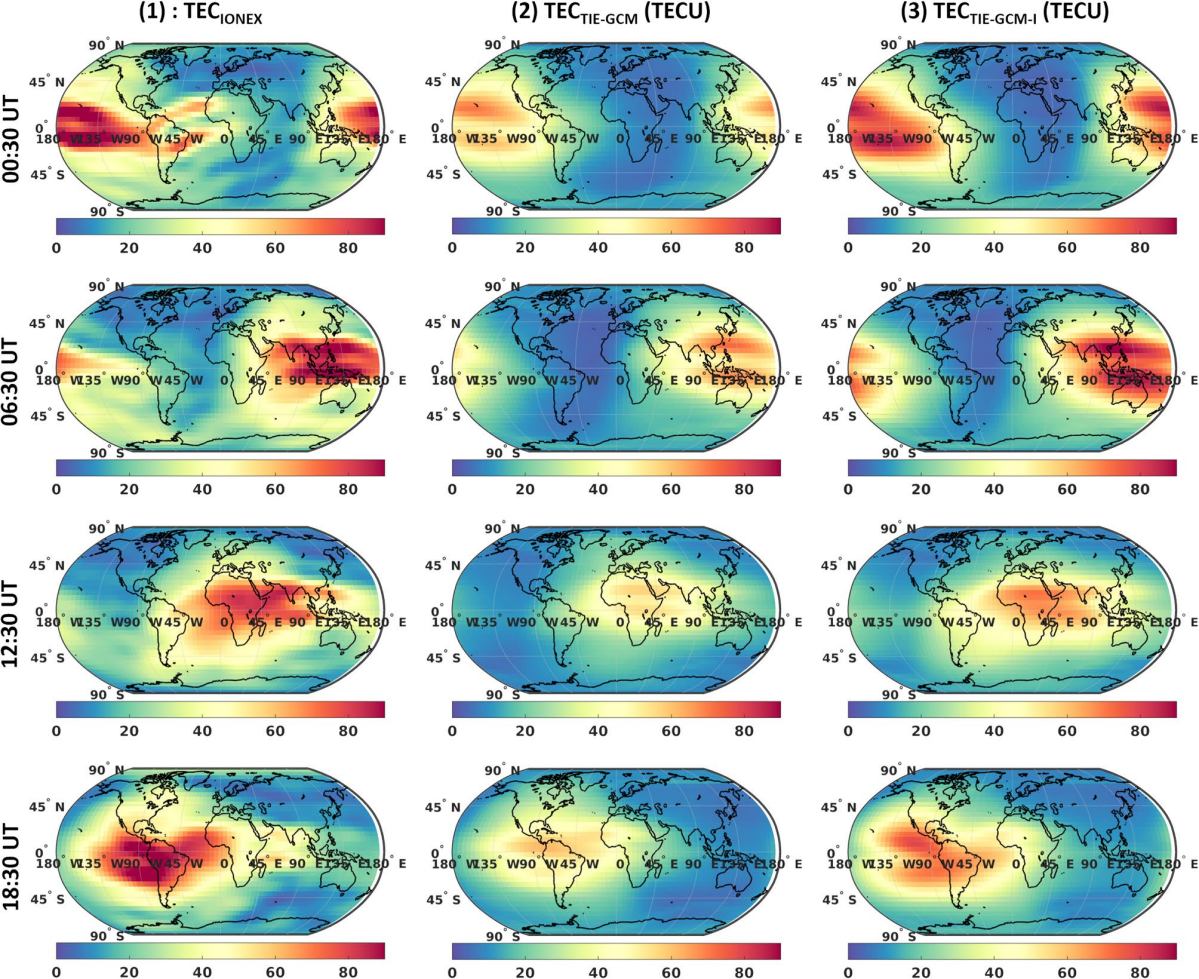
The 1st, 2nd, and 3rd columns present the TEC estimated derived from the IONEX final products, TIE-GCM, and TIE-GCM-I, during February 8th, 2015, respectively. MATLAB (version R2021a, https://www.mathworks.com/) was been used to generate these plots.

The effect of TIE-GCM-I in forecasting of TEC is shown here as an example during February 8th, 2015 (see Fig. 12), where from left to right, we present: (1) four snapshots of TEC from IGS IONEX, (2) TIE-GCM, (3) TIE-GCM-I. Comparison between (2) and (3) indicates that the forecasts of TEC estimates in TIE-GCM-I agree better with those of IGS (i.e., RMSE of 13.4, 13.8, 14.6 and 14.4 TECU for (2), while 11.3, 10.6, 8.1 and 10.9 TECU for (3)). The average reduction in the errors of TEC forecasts was found to be $26.6\%$. We also observe that the dynamics of TEC changes are well captured by TIE-GCM-I no matter the default patterns are homogeneous, see e.g., Fig. 12(1) at 06:00 or containing local peaks, see e.g., Fig. 12(1) at 14:00. Besides, the capability of TIE-GCM-I to influence the equatorial ionosphere anomaly (EIA)^77^ is enhanced by increasing the magnitude of TEC changes due to EIA about 22.5$\%$ on average around $\pm 30^{\circ }$ latitude at global constant local time at 12:00 LT. Note that the results for altitudes below 200 km must be interpreted with caution as the uncertainty of RO estimates of this altitude range may be considerably larger than those at higher altitudes. Besides, the C/DA results of GRACE TND are effective (at a range of 100 to 200 km) around the altitude of the mission.

**Figure 13:**
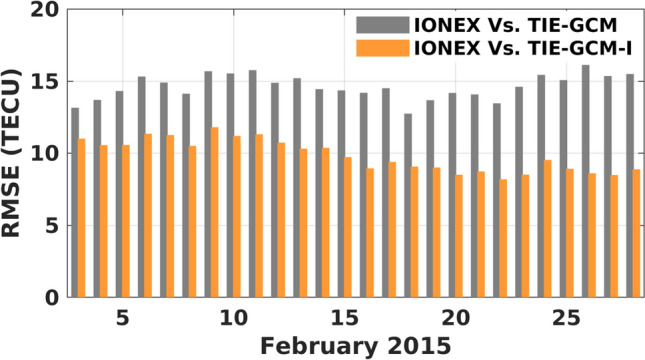
A comparison between the RMSE of TEC estimates during February 2015. Here, TEC estimates in the forecasting mode of the original TIE-GCM and TIE-GCM-I models are compared with the IONEX final TEC product from IGS. The plot was generated using MATLAB (version R2021a, https://www.mathworks.com/).

TIE-GCM-I is run to predict TEC for the entire February 2015, for which the TEC estimates from TIE-GCM-I are found to be better fitted to the IONEX final TEC products than the original TIE-GCM, see Fig. 13. Our results indicate that TIE-GCM-I can decrease the monthly average RMSE from 14.6 to 9.8 TECU and the average improvement is found to be 31.01$\%$ (in the range of 15.8-44.3$\%$). This is, in fact, a considerable improvement that is achieved by replacing the input thermosphere-related history files only, without assimilating ionosphere-related measurements.

## Conclusions

In this study, thermospheric neutral density (TND) estimates from the Gravity Recovery and Climate Experiment (GRACE) are used to re-calibrate the NRLMSISE-00’s parameters. For this, the simultaneous calibration and data assimilation (C/DA) technique^46,47^ based on the Ensemble Kalman Filter (EnKF)^78^ is implemented. This new calibrated model, C/DA-NRLMSISE-00, is then used to forecast multi-level global TND maps as well as individual neutral mass density and thermospheric temperature for the next 1 h to 21 h. The TND estimates from the European Space Agency (ESA)’s Swarm^14^ mission are used for validating the TND results. To understand the impact of the thermospheric TNDs on forecasting ionospheric variables, the output of C/DA-NRLMSISE-00 are used to replace the history files of TIE-GCM, i.e., forming an improved model TIE-GCM-I. The electron density and vertical total electron content (VTEC, i.e., simply called TEC in the entire manuscript) of TIE-GCM and TIE-GCM-I are evaluated with those from RO and GNSS measurements. Therefore, by this evaluation, one might say that the C/DA of thermosphere-related variables is validated against ionosphere-related measurements.

The main findings of this study can be summarized as:

- C/DA-NRLMSISE-00 provides TND and individual mass density estimates with smaller uncertainty than the original NRLMSISE-00 model. The TND measurements of GRACE and Swarm in both validation and forecasting steps were found to be closer to the C/DA estimates. Besides, the calibrated parameters provide the opportunity to extend the updates globally and in different altitudes. From our assessments, we found considerable changes of TND estimates for the range of 300 to 500 km. In addition, C/DA-NRLMSISE-00 is found to be more effective than the original NRLMSISE-00 to represent the equatorial thermosphere anomaly (ETA). The differences between the original and C/DA-NRLMSISE-00 indicate that the maximum relative error is reached around ETA and the increase in its magnitude is found to be around 20–30%.
- Exploring the spatial and temporal differences between the TNDs of NRLMSISE-00 and C/DA-NRLMSISE-00 using the principal component analysis (PCA) technique indicates that the daily difference between these two models in TND simulations reaches up to $10^{-12}\, \mathrm{kg}/\mathrm{m}^{3}$ and the effect of this difference on the atmospheric drag is determined about $34\%$ (on average) along typical GRACE-like satellite orbits at 400 km altitude.
- The impact of the updated mass mixing ratio (mmr) values, from C/DA-NRLMSISE-00, to be used as the primary history files of TIE-GCM (shown by TIE-GCM-I) provides a very positive impact on the global forecast of electron density and TEC. Experiments during February 8th, 2015 (i.e., a day with high solar activity) shows that the proposed method is able to decrease the errors by up to $26.48\%$ compared to the TEC estimates from IGS product (IONEX)^49^. We also compared TIE-GCM and TIE-GCM-I with RO measurements, which showed that the vertical profiles of the forecasts can be positively improved $30.92\%$ on average.
- Previous studies, dealing with the data assimilation of upper atmosphere, already demonstrated the success EnKF-based data assimilation while using TIE-GCM as base model^41,79,80^. In these studies, however, electron density was used as main observation to tune the model’s skills for estimating ionosphere related parameters. In fact, the assimilation of electron observations requires the application of localization techniques that limits their application for large scale or global case studies. The proposed C/DA instead is able to eliminate this requirement because the global input primary history files can be replaced by those of C/DA-NRLMSISE-00.
- The electron density is expected to reach its maximum at the equator because of the energy received from the Sun. The equatorial ionization anomaly (EIA^77^) is a measure that represents this fact, and can be around $\pm 30$ degree. Comparing the estimates of TEC from TIE-GCM and TIE-GCM-I at 12:00 LT for a typical day in February 2015 showed stronger anomalies after introducing the C/DA-NRLMSISE-00 input files. The differences were around $22.5\%$ of the magnitude of EIA itself, which indicates that the underestimation of neutral density mass in the original model limits its ability to represent the latitudinal electron propagation.
- Results of this study, in particular the demonstrated opportunity by implementing the complementary simultaneous data assimilation and model calibration framework, have important implications for maximizing the effective utilization of TND data in thermospheric models. Implementing the C/DA on an empirical model is computationally very efficient and fast ($\sim$111 s using 75 ensembles, 4 cores, and 10 GB RAM) thanks to the light-computational costs of NRLMSISE-00. This provides an opportunity to replace the initial values of coupled models such as TIE-GCM to reach global and multi-level thermosphere and ionosphere predictions. A similar 3-h data assimilation (using DART^81^) of TNDs that works directly for TIE-GCM takes ($\sim$4800 s for 75 ensembles, using 4 cores and 10 GB RAM). Besides, this latter implementation does not produce globally distributed and multi-level updated forecasts.

This work can be extended by performing multi-mission TND C/DA, as well as testing this technique on other types of empirical models. The advantage of the proposed method for near-real time orbit prediction and point positioning applications will be further explored.

## Supplementary Information

Supplementary Information.
