# Supplementary material for: Forecasting global and multi-level thermospheric neutral density and ionospheric electron content by tuning models against satellite-based accelerometer measurements
Source: Sci Rep. 2022 Feb 8;12:2095. doi: 10.1038/s41598-022-05952-y (PMC8825828; doi:10.1038/s41598-022-05952-y)
Supplement: Supplementary file 1 — Supplementary Information. [file 41598_2022_5952_MOESM1_ESM.pdf]

Supplementary information for 'Forecasting Global and Multi-Level Thermospheric Neutral Density and Ionospheric Electron Content by Tuning Models Against Satellite-based Accelerometer Measurements'

Ehsan Forootan[a,1], Mona Kosary[b], Saeed Farzaneh[b], Timothy Kodikara[c], Kristin Vielberg[d], Isabel Fernandez-Gomez[c], Claudia Borries[c], Maike Schumacher[a]

[a]Geodesy Group, Department of Planning, Aalborg University, Rendburggade 14, 9000, Aalborg, Denmark
[b]School of Surveying and Geospatial Engineering, College of Engineering, University of Tehran, PO.113654563, Tehran, Iran
[c]Institute of Solar-Terrestrial Physics, German Aerospace Center (DLR), Kalkhorstweg 53, 17235, Neustrelitz, Germany
[d]Institute of Geodesy and Geoinformation, University of Bonn, Nussallee 17, 53115, Bonn, Germany

1. Introduction

This document contains the following supporting information:

1. Detailed mathematical description of the calibration and data assimilation (C/DA) approach based on the ensemble Kalman filter (EnKF) to use accelerometer derived thermospheric neutral density (TND) measurements for improving NRLMSISE-00;
2. Mathematical description of the principal component analysis (PCA) method used to extract orthogonal modes from global map of TNDs;
3. Methodology of TND estimation from the Gravity Recovery and Climate Experiment (GRACE) and Swarm missions measurement;

4. Transforming TNDs to common altitudes;
5. Computing empirical covariances between the TND outputs of NRLMSISE-00 and its parameters;
6. Comparisons of the thermospheric constituents, which are obtained from TIEGCM, and the C/DA outputs (i.e., the C/DA-NRLMSISE-00 model).

2. Data

2.1 GRACE

The Gravity Recovery and Climate Experiment (GRACE, 2002-2017) (*Tapley et al.*, 2004) and its follow-on mission (GRACE-FO, launched in 2018) (*Flechtner et al.*, 2014), were designed to provide information about the Earth's gravity field. Since the orbital altitude of these satellites was low (below 550 km), these missions are equipped with accelerometer sensors to measure non-gravitational forces acting on their surface. The major contribution of the non-gravitational acceleration at low altitude is related to the atmospheric drag due to the movement of the satellite through the thermosphere. Therefore, GRACE and GRACE-FO provide measurements, which are used to estimate TND along the orbit of satellites (*Sutton et al.*, 2007; *Sutton*, 2008, 2011; *Doornbos*, 2012). In this study, the GRACE-TND is derived from an iterative estimation procedure by *Vielberg et al.* (2018). Computing TNDs requires on several data-sets and models such as: dynamic orbits of GRACE (Level 1B GNV1B Product), macro-model (*Bettadpur*, 2012), the accelerometer measurements (ACC1B), satellite's mass (MAS1B) derived from tank sensors, and the star camera data (SC1B). In a first step,

the accelerometers on-board GRACE need to be calibrated within a dynamic precise orbit determination procedure (*Vielberg et al.*, 2018). Secondly, the non-gravitational forces, which act on the surface of the satellite (i.e., consisting the Earth radiation pressure (ERP) and solar radiation pressure (SRP)) are simulated. The SRP and ERP accelerations are estimated based on the method presented in *Vielberg and Kusche* (2020). ERP and SRP accelerations are subtracted from the calibrated accelerometer measurements to obtain simulated aerodynamic accelerations. Then, the resulting aerodynamic acceleration can be used to estimate TNDs. In this study, we apply GRACE-TNDs from *Vielberg et al.* (2021) as assimilation data set.

2.2 Swarm

The three Swarm satellites were launched on November 22, 2013, into near-polar orbits at a mean altitude of 480 km (Swarm-A and -C) and 528 km (Swarm-B) (*Friis-Christensen et al.*, 2006). Each Swarm satellite carries an accelerometer and GPS receiver. These measurements can be used for determining non-gravitational forces such as drag and radiation pressure acting on the spacecraft.

In this study, the high-quality TNDs from Swarm GPS observations are used and their estimation procedure is summarized in the following (*Van Den IJssel et al.*, 2020). At first, the non-gravitational accelerations are estimated in a GPS-based precise orbit determination (POD) procedure. In this step, the range and phase information in the Swarm GPS measurements are converted to the non-gravitational accelerations based on an extended Kalman filter (EKF) approach. The gravitational forces, which are simulated, include using GOCO03s as the global Earth gravity field, FES2004 for

the ocean tides, IERS2003 conventions for the solid Earth and pole tides. Also, the accelerations due to SRP and ERP have to be subtracted from the estimated total non-gravitational accelerations. For SRP, a conical shadow model with umbra/penumbra transitions is used and then the CERES data are used to model ERP. In the second step, the direct approach (*Doornbos et al.*, 2010) is used to convert the accelerations into thermospheric densities. In this study, Swarm-TNDs from GPS data are downloaded from `http://thermosphere.tudelft.nl/` at 30-second sampling rate.

3. Transforming TNDs to Common Altitudes

To minimize the effect of differences in orbital altitudes of GRACE and Swarm missions, their TND estimates are transformed to the common altitude of 400 km using the height-dependent function based on vertical profile of NRLMSISE-00 (*Picone et al.*, 2002). The density at 400 km ($\rho(400)$) is:

$$\rho(400) = \rho(h)\,\frac{\rho_N(400)}{\rho_N(h)}, \tag{1}$$

where $\rho(h)$ is the observed density at the altitude h of GRACE and Swarm, while the subscript N stands for the NRLMSISE00 model. This transformation is applied only for performing a fair comparison between various missions. For the C/DA procedure, the TND profiles at the altitude of GRACE are used (i.e., no vertical transformation was applied).

4. Empirical Covariance Matrix between TNDs and Parameters

In this section, the empirical covariance matrix is computed using the NRLMSISE-00 derived TNDs and 19 arbitrary parameters as example. These

parameters / inputs are likely among the most sensitive to the changes of neutral density. The estimation follows the approach in *Schumacher et al.* (2015), for which during February 2015, 90 ensemble members are generated similar to those of GSA in Section 6. The correlation coefficients (CCs) between each parameters / inputs and the grid point averaged TNDs are computed that demonstrate whether these observations will be able to calibrate the parameters. The results are shown in Fig. (S1) that correspond to the longitude 15° and different latitudes from $-90°$ to 90°. The figure shows that the highest positive and negative CCs (for $C_{F_{10.7A}}$ and C_{Ap} , $PTM(1)$ and $PT(1)$) are -55.7%, -18.3%, 42.5% and 41.8%, respectively. These highest values are associated with the four parameters, which are identified by GSA. This investigation convinces us that the parameters are correctly selected and eventually can be (re-)calibrated within the C/DA procedure.

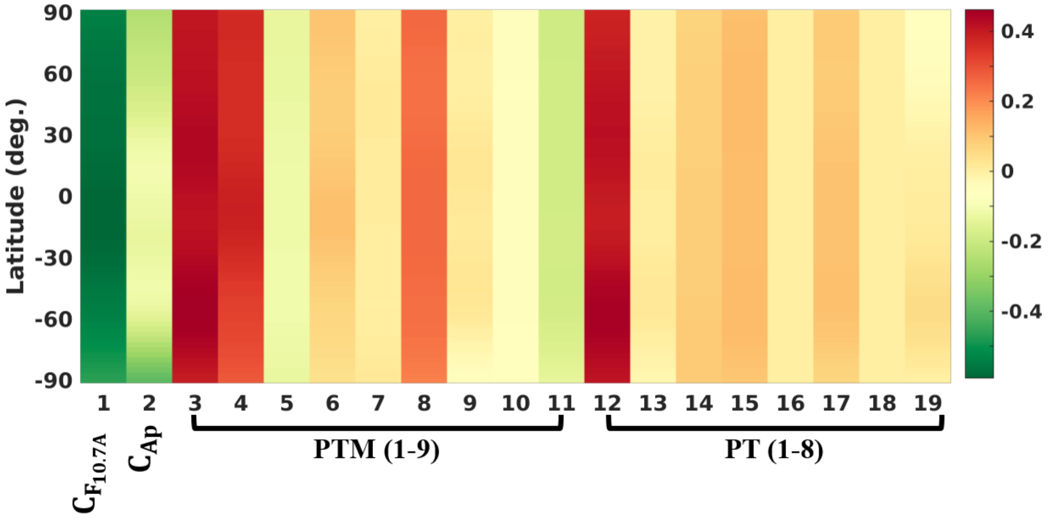

Figure S1: Averaged correlation coefficients (CCs) between 19 model parameters / inputs and TNDs obtained from the NRLMSISE-00 model during February 2015. Results correspond to arbitrary grid points with the longitude of 15° and the latitudes change from −90° to 90°. The plot was made in MATLAB (version R2021a, https://www.mathworks.com/).

5. Spatial and Temporal Differences between the C/DA-NRLMSISE-00 and TIEGCM TND Changes

The effect of replacing the improved initial history files of TIEGCM using C/DA-NRLMSISE-00 is discussed in the paper. Here, we demonstrate the variation of mass mixing ratio between TIEGCM and TIEGCM-I (TIEGCM-I is the same as TIEGCM but the mass mixing ratio of its primary history files are derived from C/DA-NRLMSISE-00. To understand how TNDs of GRACE can modify these mixing ratios horizontally and vertically, we show a comparison of the three thermospheric constituents of atomic oxygen (O), molecular oxygen (O_2) and helium (He) derived from TIEGCM and TIEGCM-I during February 8th, 2015. The results are shown in Figs. (S2

to .S5). The results indicate that the magnitude of mass mixing ratio of O, (O_2) and He changes in the range of -36.56 to 44.13%, -81.66 to 137.52% and -42.10 to 137.13% during February 8th, 2015.

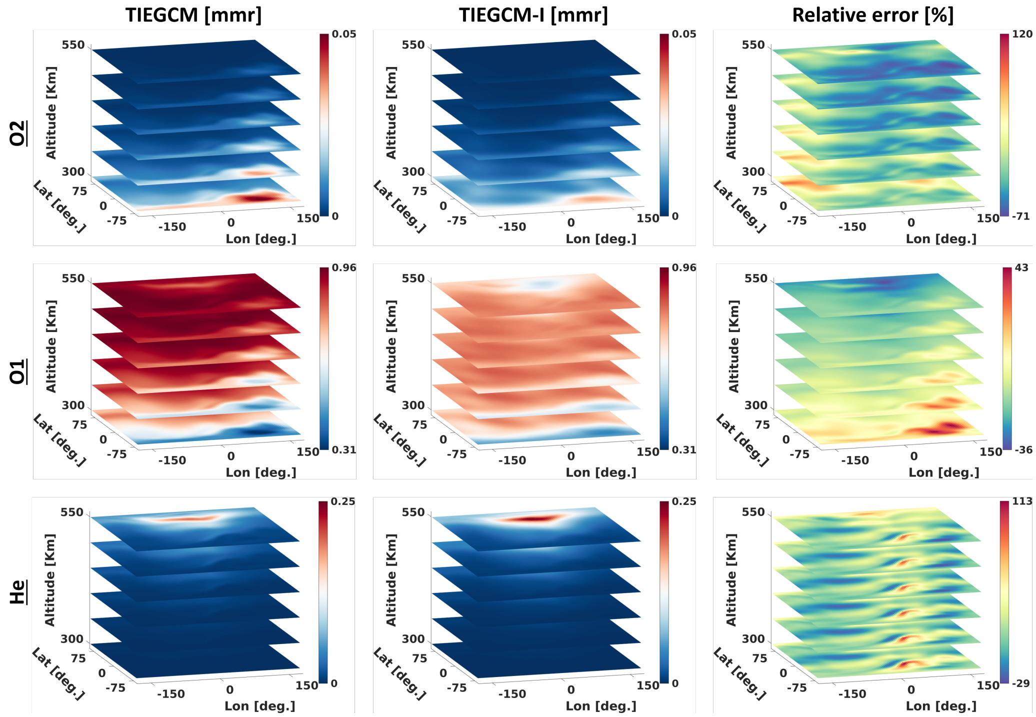

Figure S2: The 1st, 2nd, and 3rd columns present the mass mixing ratio of thermospheric constituents (i.e., atomic oxygen (O), molecular oxygen (O_2) and helium (He)) derived from TIEGCM, TIEGCM-I and the relative error (RE) estimates between these models, respectively during February 8th, 2015 at 3h UT. The plots were generated using MATLAB (version R2021a, `https://www.mathworks.com/`).

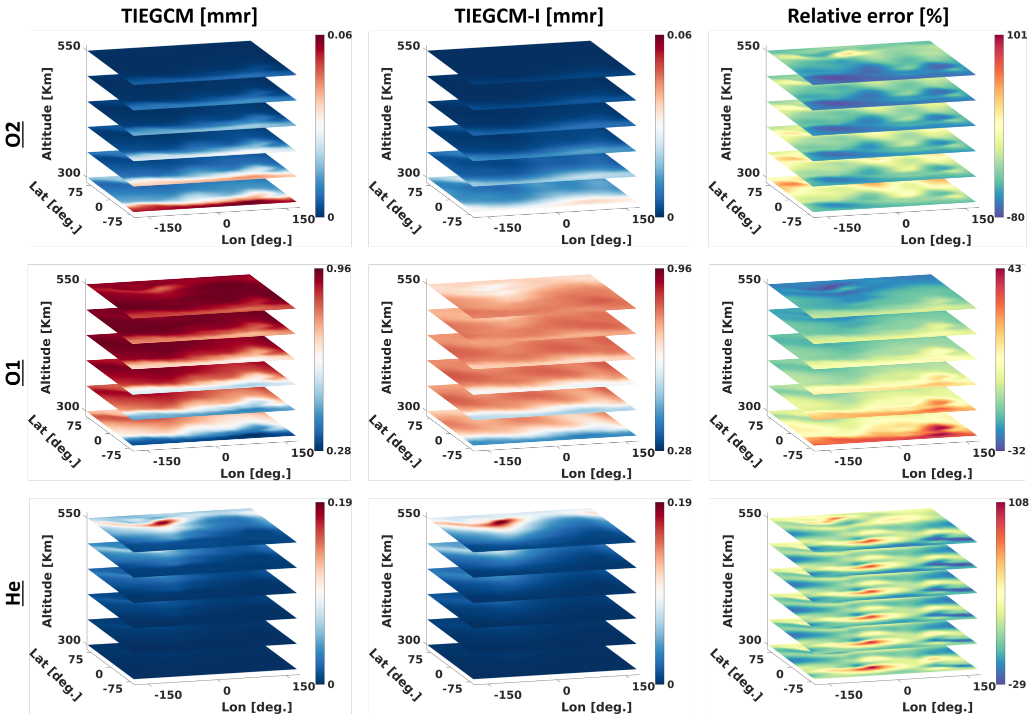

Figure S3: Similar to Fig. (S2) but at 9h UT. The plots were generated using MATLAB (version R2021a, https://www.mathworks.com/).

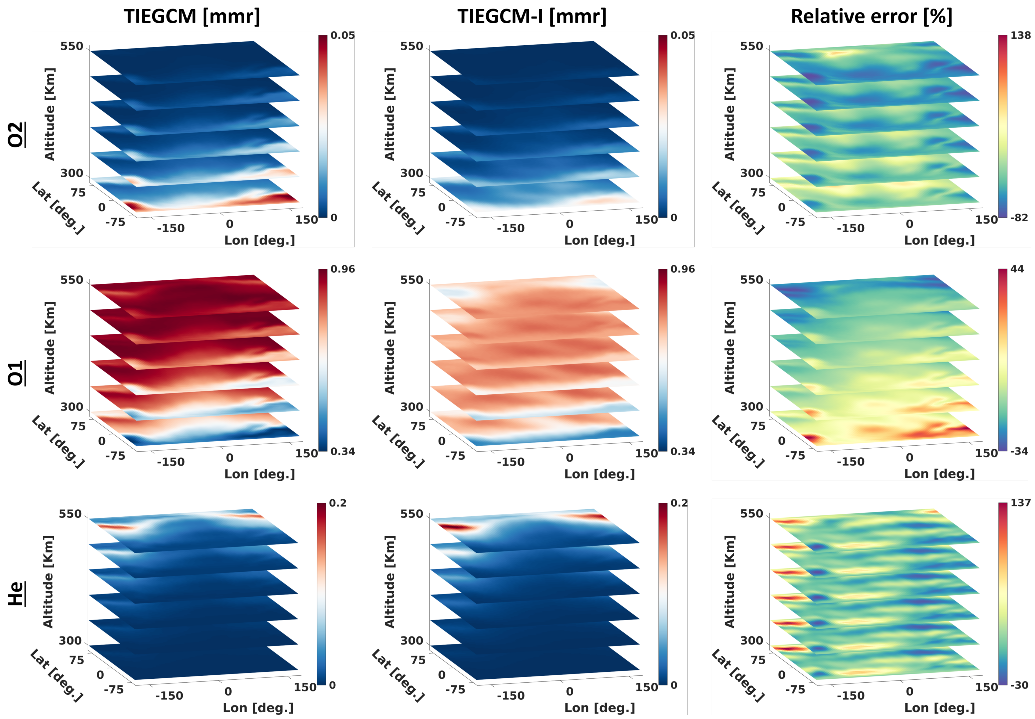

Figure S4: Similar to Fig. (S2) but at 15h UT. MATLAB (version R2021a, https://www.mathworks.com/) was used to generate these plots.

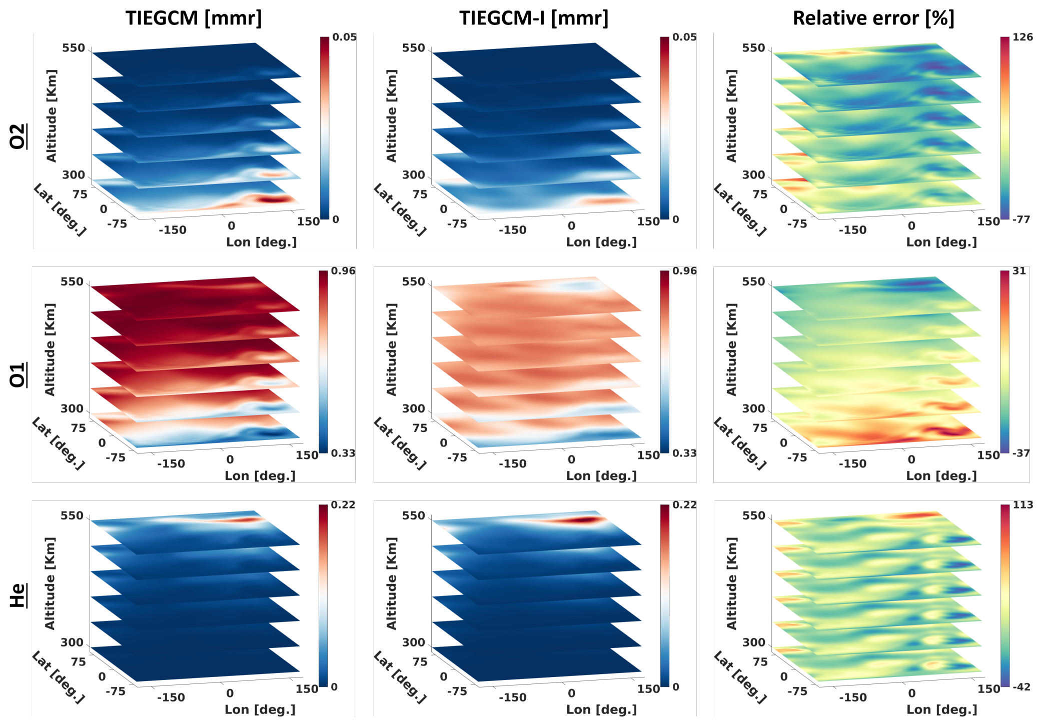

Figure S5: Similar to Fig. (S2) but at 21h UT. The plots were generated in MATLAB (version R2021a, https://www.mathworks.com/).

6. Calibration and Data Assimilation (C/DA)

The calibration and data assimilation (C/DA, *Schumacher*, 2016; *Forootan et al.*, 2020) technique is applied to tune the NRLMSISE-00 model using the TND measurements of GRACE. C/DA is applied through a model-state equation, where the model derived TNDs and some model parameters are the unknowns of this system. There are computed simultaneously in a sequential manner through minimizing the following cost function:

$$J(\mathbf{X}) = \frac{1}{2}[\mathbf{X} - \bar{\mathbf{X}}^b]^T(\mathbf{P}^b)^{-1}[\mathbf{X} - \bar{\mathbf{X}}^b] + \frac{1}{2}[\mathbf{H}\mathbf{X}^b - \mathbf{Y}]^T\mathbf{R}^{-1}(\mathbf{H}\mathbf{X}^b - \mathbf{Y}), \quad (2)$$

where $\mathbf{X}^b$ is the ensemble of model parameters and model state, $\mathbf{P}^b$ and H are the error covariance matrix of the background model and the design matrix that relates TNDs to model states and parameters. The ensemble of TND measurements is represented by Y, and R holds the uncertainty of these measurements. The details of these variables are described in the following.

At the first, it is necessary to decide which model parameters must be updated (or calibrated) within the model-state equation (Eq. 2). For this purpose, the most sensitive parameters to the TND changes are detected in this study by applying the global sensitivity analysis (GSA, *Saltelli*, 2002). GSA can identify the key parameters by examining the changes in the NRLMSISE-00's TND simulations due to the variations of its input variables and the model coefficients. Selecting a few parameters can reduce the time of model calibration and increases its efficiency by focusing the estimation efforts on the key drivers of TND estimates (*Sobol*, 1990; *Forootan et al.*, 2020).

Here, we test the impact of the following input variables and parameters on the model derived TNDs: altitude (H), geodetic latitude (φ) and longitude (λ), Local apparent Solar Time (LST), solar flux for previous day ($F10.7$) and its three-month average ($F10.7A$), 3-hourly magnetic index (Ap) as well as the model coefficients including: $pavgm$, pd, pdl, pdm, pma, ps, pt, ptl, ptm and sam. These coefficients are used to compute the number (or density) of He, O, N2, O2, Ar, H, and N, total mass density, as well as the neutral and exospheric temperature (*Picone et al.*, 2002). Since no a priory information about the NRLMSISE-00's input variable was available, GSA is implemented by generating 90 ensembles of the mentioned parameters using the Gaussian distribution with the mean equal to the default value

of these parameters and the standard deviation of 10% of their value. The GSA recognized that the first components of ptm and pt model coefficients as well as the bias of two constants of 150 and 4 in $dF10.7A = F10.7A - 150$ and $dAp = Ap - 4$ are the most sensitive parameters. Therefore, this study focuses on calibrating these four key parameters within C/DA.

The core of C/DA is selected to be the EnKF (as in *Evensen*, 2009; *Schumacher*, 2016; *Forootan et al.*, 2020). This technique uses the available measurements sequentially and based on their error covariance and those of model, it decides how to update the model states and its parameters.

First, let us assume that the original NRLMSISE-00 model is mathematically represented as:

$$\text{Original model, i.e., NRLMSISE-00} : F(\Theta) = F(\Theta_P, \Theta_R, \Theta_I), \tag{3}$$

where Θ is a vector of parameters and input values in the model. In our formulation, we consider that Θ consists of $\Theta_{P_{m_1 \times 1}}$ that are the four key parameters ($m1 = 4$) from GSA and will be updated through C/DA, Θ_R represents those parameters that will remain unchanged during the calibration, and Θ_I indicates the input variables such as the solar and geomagnetic indices, location, and time.

Ensembles of the model's key parameters are generated by a Monte Carlo simulation that considers i^{th} (i.e., $i = 1, ...n$) ensemble members of the key parameters ($\mathbf{X}^b_{1,i}$) expressed as:

$$\mathbf{X}^b_{1,i} = \Theta_P + \xi_i,\ i = 1, ...n, \tag{4}$$

where $\Theta_{P_{m_1 \times 1}}$ is a vector of default values of the key parameters in NRLMSISE-00 as in Eq. (3) plus random errors (ξ_i) that perturb these initial values. Sim-

ilar to GSA, the magnitude of noise is decided to be 10% of each variable. In the C/DA procedure, ensembles of 75 members ($n = 75$) are used to perform the numerical integration. The 5 minutes TNDs from GRACE orbit which makes $m_2 = 30$ observations are used in each step of C/DA.The assimilation window is selected to be three hours, because the minimum RMSE between C/DA-NRLMSISE-00 and Swarm-A, -B, and -C is obtained from calibrated parameters of this window.

The assimilation window size is determined experimentally by changing its size from 1 hour to 5 hours. In each experiment, the calibrated parameters were used to predict the TNDs of the next hours. The minimum RMSE (Eq. 8), against Swarm-A, -B, and -C TNDs, was found to be related to the window size of 3 hours.

The ensemble of key parameters ($\mathbf{X}_1^b$) and model states (i.e., simulated TNDs using perturbed key parameters ($\mathbf{X}_2^b$=$F(\Theta_P + \xi, \Theta_R, \Theta_I)$) are stored in the matrix $\mathbf{X}_{m\times n}^b$ as:

$$\mathbf{X}^b = \begin{bmatrix} \mathbf{X}_{1_{m_1\times n}}^b \\ ------ \\ \mathbf{X}_{2_{m_2\times n}}^b \end{bmatrix}, \tag{5}$$

where the upper-index 'b' represents the background model. The ensemble mean vector ($\bar{\mathbf{x}}_{m\times 1}^b$) of Eq. (5) and the covariance matrix of the background step ($\mathbf{P}_{m\times m}^b$) are defined as:

$$\bar{\mathbf{x}}^b = \begin{bmatrix} \bar{\mathbf{x}}_1^b \\ \bar{\mathbf{x}}_2^b \end{bmatrix}, (e.g.,\ \bar{\mathbf{x}}_1^b = \frac{1}{n}\sum_{i=1}^{n} \mathbf{x}_{1,i}^b), \tag{6}$$

$$\mathbf{P}^b = \frac{1}{n-1}(\mathbf{X}^b - \bar{\mathbf{x}}^b)(\mathbf{X}^b - \bar{\mathbf{x}}^b)^T. \tag{7}$$

In each analysis step, shown by the upper-index 'a', the estimation of key parameters and the model state ($\mathbf{X}^a$) follows:

$$\mathbf{X}_{m\times n}^a = \mathbf{X}^b + \mathbf{K}(\mathbf{Y} - \mathbf{H}\mathbf{X}^{\mathrm{b}}), \tag{8}$$

and their ensemble mean, shown by $\bar{\mathbf{x}}^a$, is computed as:

$$\bar{\mathbf{x}}_{m\times 1}^a = \bar{\mathbf{x}}^b + \mathbf{K}(\bar{\mathbf{y}} - \mathbf{H}\bar{\mathbf{x}}^b). \tag{9}$$

Here, $\mathrm{Y}_{m_2\times n}$ and $\bar{\mathbf{y}}_{m_2\times 1}$ represent the ensembles and the ensemble mean of GRACE-TNDs, respectively. Therefore, according to Eqs. (8 and 9), the updates of key parameters and model states directly depend on the differences between the real observations (Y) and model predictions ($\mathbf{H}\mathbf{X}^{\mathrm{b}}$), while considering their weights, which are reflected in the Kalman gain matrix ($\mathbf{K}_{\Theta m\times m_2}$) that is computed as:

$$\mathbf{K} = \mathbf{P}^{\mathrm{b}}\mathbf{H}^{\mathrm{T}}\left(\mathbf{H}\mathbf{P}^{\mathrm{b}}\mathbf{H}^{\mathrm{T}} + \mathbf{R}\right)^{-1}, \tag{10}$$

where $\mathbf{H}$ is design matrix, which is defined as:

$$\mathbf{H}_{m_2\times m} = [\mathbf{0}_{m_2\times m_1}\ \ \mathbf{I}_{m_2\times m_2}], \tag{11}$$

where $\mathbf{0}_{m_2\times m_1}$ is a zero matrix, and $\mathbf{I}_{m_2\times m_2}$ represents the identity matrix. This means that in each step of the Kalman Filter process, the relationship between observations and model states is assumed to be linear.

The C/DA procedure (Eq. 5 to Eq. 11) has been evaluated at each time step to obtain the ensemble of parameters and states (i.e., $\mathbf{X}^a$), and their mean (i.e., $\bar{\mathbf{x}}^a$). Analogous to Eqs. (5 and 6), $\mathbf{X}^a$ and $\bar{\mathbf{x}}^a$ are divided into two section as:

$$\mathbf{X}^a = \begin{bmatrix} \mathbf{X}^a_{1_{m_1 \times n}} \\ ------ \\ \mathbf{X}^a_{2_{m_2 \times n}} \end{bmatrix}, \text{and} \qquad \mathbf{x}^a = \begin{bmatrix} \mathbf{x}^a_{1_{m_1 \times n}} \\ ------ \\ \mathbf{x}^a_{2_{m_2 \times n}} \end{bmatrix}, \tag{12}$$

where $\mathbf{X}^a_1$ and $\mathbf{x}^a_1$ contain the ensembles of model parameters and their mean that are estimated in the analysis step.

The ensemble of key parameters from the analysis step ($\mathbf{X}^a_1$) is used for the background step ($\mathbf{X}^b_1$) of the next time step in simulating TNDs values and the C/DA procedure continues until the observations are available.

The C/DA procedure is performed using 3 hours of GRACE-TNDs. The last set of key parameters that are estimated are considered as the optimal solution, which provides us with $\hat{\Theta}_P$ in Eq. (13). These parameters then replace the default values of the original NRLMSISE-00 model Eq. (3) to now-cast and forecast (for the next hour) multi-level TNDs, individual neutral mass densities, and thermospheric temperature globally. The C/DA model, i.e., called 'C/DA-NRLMSISE-00', is represented by:

$$\text{C/DA model, i.e., } : \mathrm{F}(\hat{\Theta}_P, \Theta_R, \Theta_I), \tag{13}$$

7. Principal Component Analysis (PCA)

Principal component analysis (PCA, *Preisendorfer*, 1988) is a statistical decomposition technique, which is applied here to extract dominant variance

of multivariate data sets. PCA works based on the eigenvalue decomposition of the auto-covariance matrix built on the data matrices. Implementation of the PCA of this study follows the one in *Forootan* (2014).

Our data sets, which can be grid maps of TND or thermospheric species, consist of m time epoch and n grid points which arranged into an m by n data matrix (i.e., $R = (\mathbf{r}_1, \ldots, \mathbf{r}_n)$). The temporal mean of data set is $\bar{\mathbf{y}}_{1,p} = \frac{1}{m}\sum_{i=1}^{m} \mathbf{r}_{i,p}$ where $p = 1, \ldots, n$, which is a raw with dimension n, and each element of $\bar{\mathbf{y}}$ is the mean value of all m observations for a given grid point.

The deviations of all observation from the mean are arranged into an $m \times n$ matrix, $\mathbf{Y} = [\mathbf{y}'_1, \mathbf{y}'_2, \ldots, \mathbf{y}'_n]$, where each column of $\mathbf{Y}$ is the deviation of one grid point of observation sample from the data set mean $\bar{\mathbf{y}}$ (i.e., $\mathbf{y}'_i = \mathbf{r}_i - \bar{\mathbf{y}}_{1,i}$) and each row of $\mathbf{Y}$ contains n observation deviations at each time epoch. The covariance matrix $\mathbf{C}$ of matrix $\mathbf{Y}$ can be written as:

$$\mathbf{C} = \frac{1}{m}\mathbf{Y}\mathbf{Y}^T, \tag{14}$$

where the superscript T is a transpose operator. Through the eigenvalue decomposition procedure, the covariance matrix $\mathbf{C}$ can be decomposed as:

$$\mathbf{C}\mathbf{E} = \mathbf{E}\Lambda^2, \tag{15}$$

where Λ is a diagonal matrix with all the eigenvalues λ_i of $\mathbf{C}$ arranged according to their magnitude, and $\mathbf{E} = [e_1 \ldots e_m]$ is an orthogonal matrix consists of corresponding eigenvectors of $\mathbf{C}$ as column vectors, where $\mathbf{E}^T\mathbf{E} = I$ and I is the identity matrix. The matrix $\mathbf{E}$ is called empirical orthogonal function (EOF), which contains the spatially orthogonal vectors. The time series that

is called principal components (PC) is estimated based on the projection of the data anomalies $\mathbf{Y}$ on the orthogonal eigenvectors $\mathbf{E}$, which means

$$\mathbf{P} = \mathbf{Y}\mathbf{E}, \tag{16}$$

and the PCA decomposition is written as:

$$\mathbf{Y} = \mathbf{P}\mathbf{E}^T. \tag{17}$$

References

Bettadpur, S. (2012), GRACE, gravity recovery and climate experiment, product specification document. rev. 4.5, *Tech. rep.*, CSR-GR-03-02, ftp://podaac. jpl. nasa. gov/allData/grace/docs/ProdSpecDoc_v4 ....

Doornbos, E. (2012), *Thermospheric density and wind determination from satellite dynamics*, Springer Science & Business Media, doi:http://resolver.tudelft.nl/uuid:33002be1-1498-4bec-a440-4c90ec149aea.

Doornbos, E., J. Van Den Ijssel, H. Luhr, M. Forster, and G. Koppenwallner (2010), Neutral density and crosswind determination from arbitrarily oriented multiaxis accelerometers on satellites, *Journal of Spacecraft and Rockets*, *47*(4), 580–589, doi:https://doi.org/10.2514/1.48114.

Evensen, G. (2009), The ensemble Kalman filter for combined state and parameter estimation, *IEEE Control Systems Magazine*, *29*(3), 83–104, doi:10.1109/MCS.2009.932223.

Flechtner, F., P. Morton, M. Watkins, and F. Webb (2014), Status of the GRACE follow-on mission, in *Gravity, geoid and height systems*, pp. 117–121, Springer.

Forootan, E. (2014), Statistical signal decomposition techniques for analyzing time-variable satellite gravimetry data, *PhD Thesis, University of Bonn*, p. 131pp, doi:http://hss.ulb.uni-bonn.de/2014/3766/3766.htm.

Forootan, E., S. Farzaneh, M. Kosary, M. Schmidt, and M. Schumacher (2020), A simultaneous calibration and data assimilation (C/DA) to improve nrlmsise00 using thermospheric neutral density (TND) from space-borne accelerometer measurements, *Geophysical Journal International*, *224*(2), 1096–1115, doi:10.1093/gji/ggaa507.

Friis-Christensen, E., H. Lühr, and G. Hulot (2006), Swarm: A constellation to study the Earth's magnetic field, *Earth, planets and space*, *58*(4), 351–358, doi:https://doi.org/10.1186/BF03351933.

Picone, J., A. Hedin, D. P. Drob, and A. Aikin (2002), NRLMSISE-00 empirical model of the atmosphere: statistical comparisons and scientific issues, *Journal of Geophysical Research: Space Physics (1978–2012)*, *107*(A12), SIA–15, doi:10.1029/2002JA009430.

Preisendorfer, R. (1988), *Principal component analysis in meteorology and oceanography*, Elsevier, Amsterdam, ISBN:0444430148.

Saltelli, A. (2002), Sensitivity analysis for importance assessment, *Risk analysis*, *22*(3), 579–590, doi:10.1111/0272-4332.00040.

Schumacher, M. (2016), Methods for assimilating remotely-sensed water storage changes into hydrological models, Ph.D. thesis, University of Bonn.

Schumacher, M., A. Eicker, J. Kusche, H. M. Schmied, and P. Döll (2015), Covariance analysis and sensitivity studies for GRACE assimilation into WGHM, in *IAG 150 Years*, pp. 241–247, Springer, doi:https://doi.org/10.1007/1345_2015_119.

Sobol, I. M. (1990), On sensitivity estimation for nonlinear mathematical models, *Matematicheskoe modelirovanie*, *2*(1), 112–118.

Sutton, E. (2008), Effects of solar disturbances on the thermosphere densities and winds from CHAMP and GRACE satellite accelerometer data, Ph.D. thesis, University of Colorado, Boulder.

Sutton, E. (2011), Accelerometer-derived atmospheric densities from the CHAMP and GRACE satellites: version 2.3, *AFRL Technical Memo, DTIC# ADA537198.*

Sutton, E., R. Nerem, and J. Forbes (2007), Density and winds in the thermosphere deduced from accelerometer data, *Journal of Spacecraft and Rockets*, *44*(6), 1210–1219, doi:10.2514/1.28641.

Tapley, B. D., S. Bettadpur, J. C. Ries, P. F. Thompson, and M. M. Watkins (2004), GRACE measurements of mass variability in the Earth system, *Science*, *305*(5683), 503–505, doi:10.1126/science.1099192.

Van Den IJssel, J., E. Doornbos, E. Iorfida, G. March, C. Siemes, and O. Montenbruck (2020), Thermosphere densities derived from Swarm

GPS observations, *Advances in Space Research*, *65*(7), 1758–1771, doi: https://doi.org/10.1016/j.asr.2020.01.004.

Vielberg, K., and J. Kusche (2020), Extended forward and inverse modeling of radiation pressure accelerations for LEO satellites, *Journal of Geodesy*, *94*(4), 1–21, doi:10.1007/s00190-020-01368-6.

Vielberg, K., E. Forootan, C. Lück, A. Löcher, J. Kusche, and K. Börger (2018), Comparison of accelerometer data calibration methods used in thermospheric neutral density estimation, *Annales Geophysicae*, *36*(3), 761–779, doi:10.5194/angeo-36-761-2018.

Vielberg, K., C. Lück, A. Corbin, E. Forootan, A. Löcher, and J. Kusche (2021), TND-IGG RL01: Thermospheric neutral density from accelerometer measurements of GRACE, CHAMP and Swarm, doi:10.1594/PANGAEA.931347.
